# Adiabatic-Bias Molecular Dynamics Simulations Reveal the Impact of Mutations on Muscarinic Antagonist Unbinding Kinetics

**DOI:** 10.1021/acs.jcim.5c00601

**Published:** 2025-06-16

**Authors:** Adriana Coricello, Anna Lisa Chiaravalle, Maria Musgaard, Benjamin Gerald Tehan, Gian Marco Elisi, Giovanni Bottegoni

**Affiliations:** † Department of Biomolecular Sciences, 19044 Università degli Studi di Urbino Carlo Bo, Piazza Rinascimento 6, 61029 Urbino, Italy; ‡ 464286 OMass Therapeutics Ltd., Building 4000, John Smith Dr, Oxford Business Park, ARC, OX4 2GX Oxford, U.K.; § Department of Pharmacy, University of Birmingham, Edgbaston, B15 2TT Birmingham, U.K.

## Abstract

Ligand–target dissociation rates (*k*_off_) strongly correlate with efficacy and safety profiles, as well as with the therapeutic effect of drugs. As a prototypical example, muscarinic receptor antagonists used as bronchodilators show similar affinity profiles toward the muscarinic M3 receptors (M3R) and M2 receptors (M2R), whereas their kinetic selectivity toward M3R avoids the adverse effects that a prolonged inhibition of M2R would induce at the cardiac level. Previous studies on the dissociation kinetics of human M3R showed that the residence time and binding affinity of muscarinic antagonists are deeply affected by the presence of specific mutations. The aim of our work was to reproduce the rankings of these experimental kinetic rates through an approach based on the application of adiabatic-bias molecular dynamics (ABMD) simulations using Path Collective Variables (PCVs), PCV-ABMD. Employing this methodology, we simulated the translocation of tiotropium, a long-acting bronchodilator targeting M3R, from the orthosteric site to the extracellular vestibule, without considering the whole unbinding process. The estimated times necessary for translocation displayed a strong correlation with the experimental p*k*_off_ values. Moreover, a thorough analysis of protein–ligand contacts provided deeper insights into the mechanism of unbinding of muscarinic antagonists. The newly described PCV-ABMD protocol captured relevant metastable states and offered a reliable approach for the prediction of kinetic selectivity in sets of mutants.

## Graphical abstract

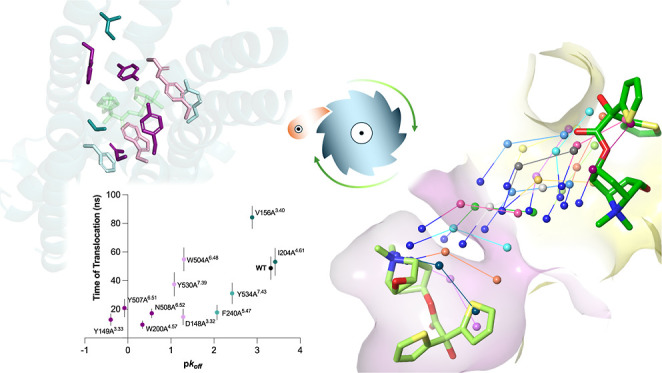

## Introduction

Advancing clinically relevant compounds to the late stages of drug discovery programs requires the optimization of multiple parameters. Among others, effective target engagement and an optimal pharmacokinetic profile are two key features that an optimized compound must possess. Residence time (RT) is a fundamental and informative criterion to predict and rationalize the duration of the biological activity and the in vivo efficacy of new compounds.^1–4^ The stability of the receptor–ligand complex correlates with the modulation of kinetic rates,^5,6^ which leads to prolonged on-target activity, thereby lowering the required ligand concentration for the activity.^6,7^ Modulation of the RT on the therapeutic profile has been particularly evidenced for drugs targeting membrane receptors such as G protein-coupled receptors (GPCRs).^8^ Often, modern drug discovery campaigns begin by computationally prioritizing a few compounds based on estimated binding affinities. However, incorporating predictions of residence time alongside affinity could ultimately yield improved in vivo efficacy during lead optimization.^6,9^

Muscarinic receptor antagonists offer a prototypical example for the impact of residence time on biological activity.^10^ Although complexes of muscarinic subtype receptors M2 and M3 (Figure 1) with tiotropium are characterized by a rather similar binding affinity (p*K*_i_ = 10.69 vs p*K*_i_ = 11.02),^11^ the slower dissociation of the ligand from M3R (*k*_off_ = 4.8 × 10^–4^ min^–1^) results in a prolonged receptor activity relative to M2R (*k*_off_ = 3.3 × 10^–3^ min^–1^). Such behavior leads to kinetic selectivity^10^ when considering the evaluation of tiotropium’s pharmacological profile as a bronchodilator.^12,13^ Indeed, thanks to its longer half-life, tiotropium can be dosed with less frequency than the other structurally related and clinically approved peripheral muscarinic antagonist, ipratropium, resulting in less severe side effects.^12,13^ Moreover, other noteworthy receptor systems, e.g., adenosine^14,15^ and melatonin^16,17^ receptors, also display distinct kinetic rates among their receptor subtypes.

**Figure 1:**
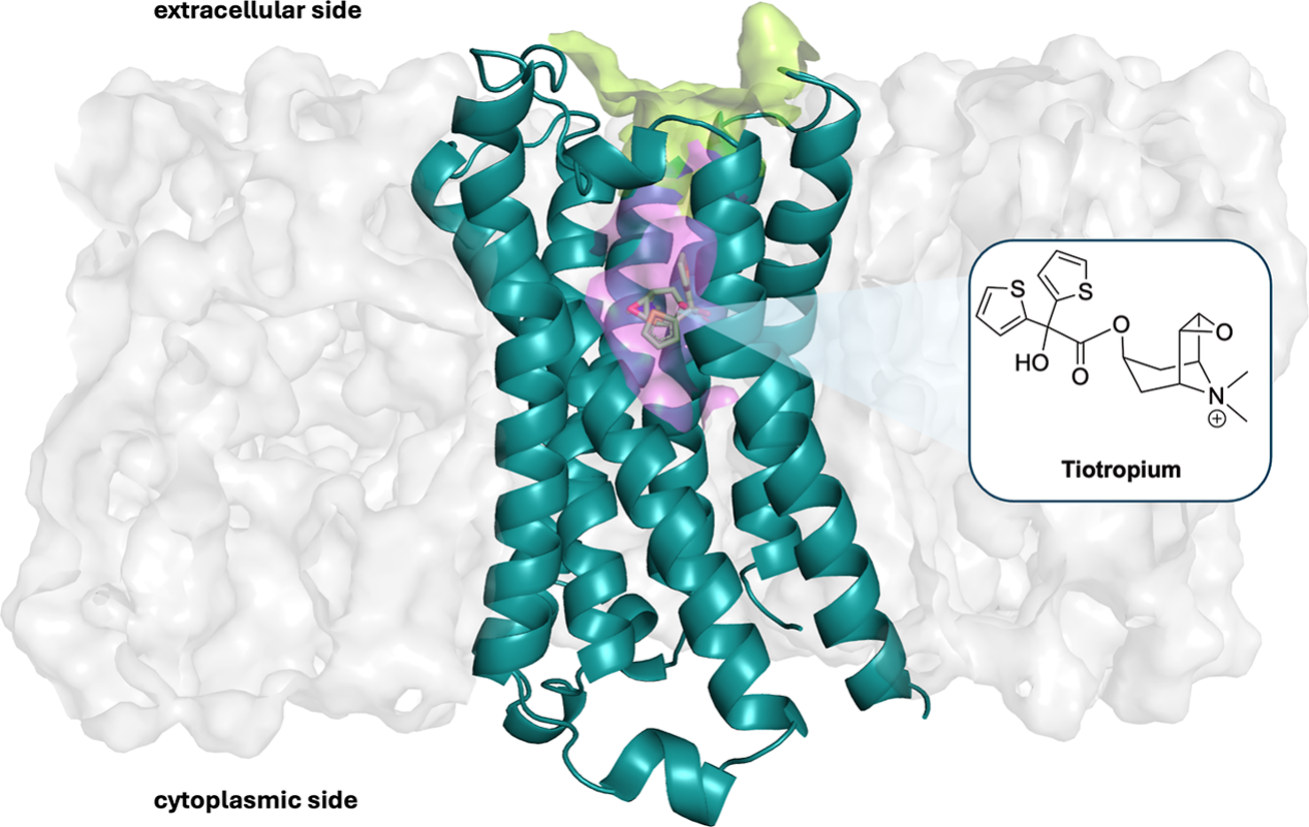
Representation of M3R (PDB ID 4DAJ)^18^ embedded in the membrane bilayer (gray surface). The protein backbone is represented as teal cartoon, and tiotropium is shown as green sticks. The orthosteric and vestibular sites are depicted as violet and yellow faded surfaces, respectively. The 2D representation of tiotropium is shown as a zoomed-in section of the co-crystallized ligand.

In 2015, Tautermann et al. reported a study based on a series of ligands and M3R mutants to dissect the structure–activity relationships (SARs) and structure–kinetics relationships (SKRs) of M3R antagonists and tiotropium derivatives and thus describe the role of functional groups in the modulation of RTs.^10^ Understanding the determinants at the basis of the binding kinetics of M3R antagonists acquired major importance considering the revived interest in the modulation of muscarinic receptors. The renewed attention for this receptor system is devoted to chronic obstructive pulmonary disease (COPD) treatment,^19^ including the design of dual-acting compounds, additionally acting as adrenergic agonists.^20^

For GPCR ligands, dissociation constants (*k*_off_) are typically determined via competitive radioligand binding experiments, in which a strong labeled binder is displaced.^21,22^ In the following case studies, these experiments were used to dissect the impact of mutations on ligand RT. Notably, Tautermann’s work focused on the effects of mutations on M3R antagonists binding kinetics, as RT modulation can significantly impact a drug’s therapeutic profile. In turn, the impact of mutations on dissociation kinetics can influence the receptor signaling bias, i.e., whether GPCR-mediated signaling preferentially evokes the G-protein pathway or the β-arrestin one.^23,24^ A study by Wacker et al. reported how single-point mutations can affect ligand’s functional efficacy: LSD interacting with mutated forms of serotonin receptors 5-HT_2A_ and 5-HT_2B_ resulted in a decreased recruitment of β-arrestin.^24^ In another study, mutations were employed to assess changes in the kinetics of structurally diverse C–C chemokine receptor type 5 (CCR5) allosteric modulators and highlighted which specific interactions, when missing, would induce selective recognition of alternative ligands.^25^

When GPCRs are involved and kinetic aspects gain prominence, simple static methods such as docking and pharmacophore searches, typically used in the earliest stages of a drug discovery campaign, give way to physics-based approaches such as molecular dynamics (MD) simulations. MD simulations afford richer insights and, moreover, recent major advances in MD-based methods significantly decreased the computational cost of these simulations.^26–28^ Among those, several protocols were purposely tailored to GPCRs with the goal of evaluating the ligand unbinding kinetics. Various biasing techniques have been exploited, including metadynamics,^29–33^ steered MD,^34^ supervised MD,^35^ and random-accelerated MD calculations.^36^ Recently, Buigues et al. employed an adaptation of the finite temperature string method^37,38^ to characterize transition states during the unbinding of M3R antagonists.^39^ To this end, protocols that bias the sampling of conformational space toward the exploration of cleverly selected path collective variables (PCVs)^40^ have been shown to enhance the characterization of intermediate metastable states compared to similar protocols employing simpler geometric variables, while also providing a more accurate description of kinetic rates.^41–43^

Several computationally intensive free-energy methods have demonstrated high quantitative accuracy in predicting residence times (RT).^41,43,44^ Recently, building on previous approaches, such as conformational flooding,^45^ hyperdynamics,^46^ and infrequent metadynamics,^47^ OPES flooding simulations were also applied to recover ligand unbinding kinetic rates, without biasing the transition state.^48,49^ Conversely, other approaches, that do not aim at calculating the absolute RT, were designed or adapted to reproduce the experimental ranking of relative RTs in pharmaceutically relevant systems with reduced computational cost.^29,33,50–56^ The core idea is that, especially within a congeneric series of compounds, a correlation exists between the experimental RT and the amount of simulated time, usually averaged over multiple replicas, required to observe an unbinding event.

Adiabatic-bias molecular dynamics (ABMD)^57^ allows for the transition of free-energy barriers along a specific reaction coordinate during MD simulations. Once an end point state has been chosen, a harmonic biasing potential is applied. As the simulation proceeds, the bias potential is applied only when the system attempts to recede along the coordinate. Thus, exploration is driven toward the predefined end point with minimal perturbation and according to thermal fluctuations of the system. Herein, we propose a protocol in which initial unbinding simulations are retrieved through preliminary ABMD simulations^57^ performed applying a simple distance collective variable (CV). Subsequently, these trajectories are used to define PCVs^40^ to improve the qualitative description of ligand unbinding and to optimize the correlation with experimental data through the use of shorter simulations. PCVs consist of two collective variables: one describing the progress along a set of predefined equidistant milestones ( S ) and the other one measuring the distance of the instantaneous position of the system with respect to the predefined path ( Z ). The latter can be restrained with a repulsive potential, concentrating simulation sampling on the relevant phase space and enriching the exploration of metastable states occurring along the unbinding pathway.

While previous computational studies focused on M3R antagonists,^29,33,39^ we turned our attention on the kinetic selectivity of tiotropium in the context of the mutations at the orthosteric site of M3R (Figure 2A) discussed by Tautermann et al.^10^ Our goal was to develop a protocol capable of retrospectively reproducing experimental relative residence times through unbinding simulations of single-point mutants, with the potential for future adaptation into a predictive tool. Our focus on the influence of mutations on ligand RTs represents a relatively new application of computational methods to assess mutational effects on kinetic rates. Such studies are typically conducted using different ligands, with a few notable exceptions, including examining the impact of mutations using τ-RAMD simulations.^58,59^

**Figure 2:**
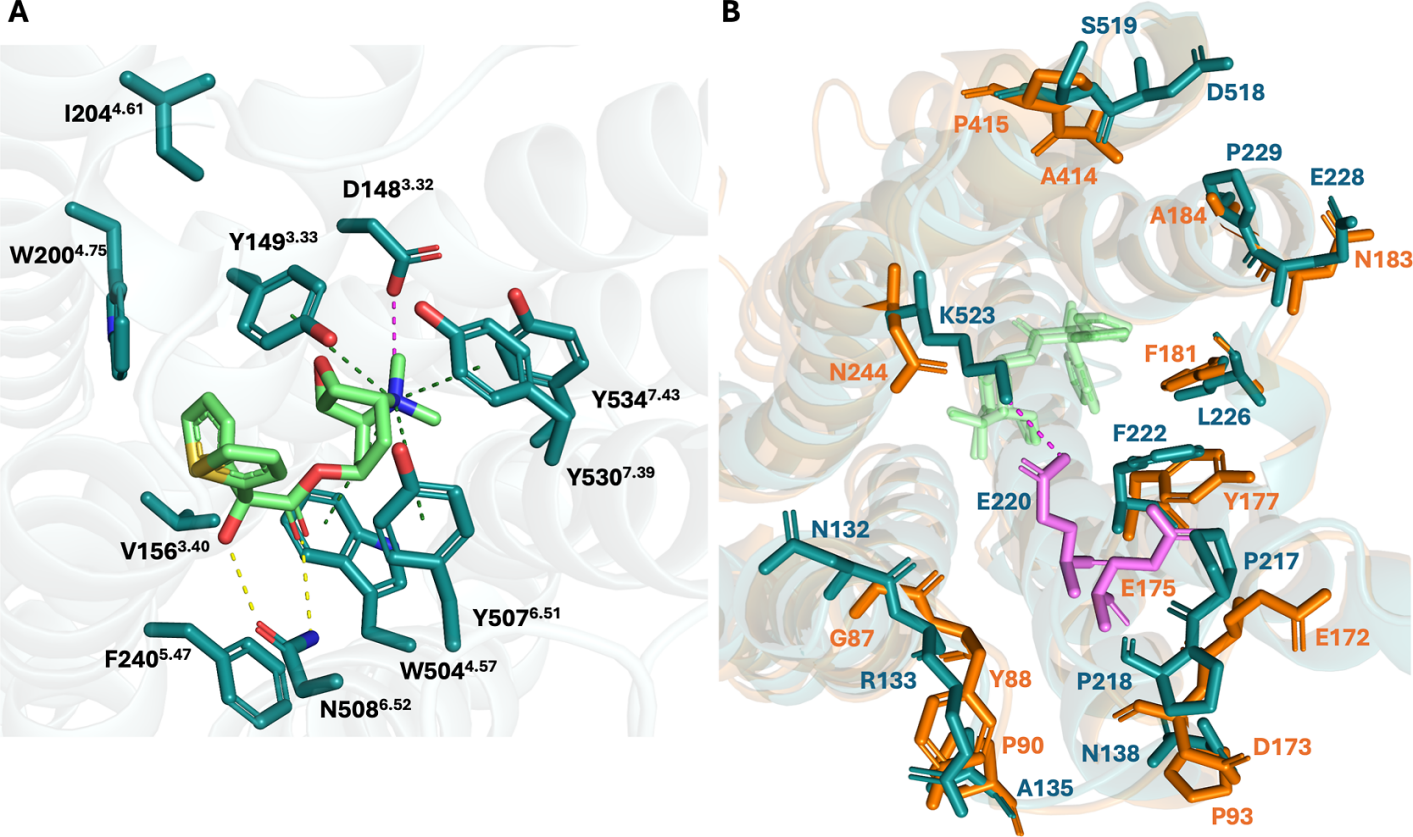
(A) Representation of the orthosteric sites residues of M3R (PDB ID 4DAJ)^18^ which were mutated in the study by Tautermann and colleagues.^10^ The protein backbone is represented as faded teal cartoon, and residues and co-crystallized tiotropium are represented as teal and light green sticks, respectively. Hydrogen bonds, salt bridges, and π–cation interactions are represented as yellow, magenta, and green dashed lines, respectively. (B) Representation of the extracellular loop region of superimposed M3R and M2R (PDB ID 4DAJ and 3UON).^18,61^ Different residues are represented as teal and orange sticks for M3R and M2R, respectively. Protein backbones are represented as faded teal (M3R) and orange (M2R) cartoons. Conserved E220/175^ECL2^ is represented as violets sticks, and tiotropium as green sticks. The salt bridge between K523^7.32^ and E220 in M3R is represented as a magenta dashed line.

In this study, we also performed a comparative analysis of unbinding of tiotropium from M3R and M2R, which have several differing residues in the extracellular loop (ECL) region but share a largely conserved orthosteric site (Figures 2B and S1). Both receptors present a similar interaction pattern with tiotropium and its analogues, including the salt-bridge interaction between the ammonium group and D148^3.32^, and a bidentate hydrogen bond between N508^6.52^ and the α-hydroxyacetyl portion^10,18^ (with the Ballesteros–Weinstein numbering scheme^60^ reported in superscript notation). Notably, only a single residue directly facing the ligand differs between the two receptors.

Adopting PCV-ABMD simulations, we assume that the translocation to the vestibular site might be sufficient to correlate unbinding times with experiments without considering the overall unbinding process. Indeed, ligand translocation from the orthosteric site to the extracellular vestibule should correspond to the main free-energy barrier preventing ligand dissociation and necessitating the opening of the “tyrosine cage”, formed by Y149^3.33^, Y507^6.51^, and Y530^7.39^. This cage is stabilized by hydrogen bonds between the hydroxyl groups of these residues.^10,18^

Understanding the transition states governing ligand dissociation may indeed be crucial for elucidating the basis for the different dissociation rates in certain mutants. In this work, we performed a comparative analysis of the unbinding mechanisms of WT-M3R and 11 orthosteric site mutants, as well as WT-M2R. The latter offered further insights into the molecular determinants underlying kinetic selectivity among muscarinic receptors.

## Results

### Preliminary Evaluation of M3 Receptor Mutant Kinetics

We initially performed 500 ns of plain MD simulations on three systems bound to tiotropium: wild-type human M3 receptor (WT M3R) to establish a baseline, Y149A^3.33^ M3R mutant, selected because this is the mutant displaying the shortest experimental half-life (approximately 17 s), and wild-type human M2 receptor (M2R, experimental half-life equal to 3.6 h). Our results did not reveal any outstanding difference in the behavior of the three systems. The analysis of the distance between the tiotropium quaternary nitrogen atom and the D148^3.32^ γ-carbon atom showed that the ionic bridge remained stable throughout the 500 ns simulations of WT-M3R. The distance peaks at 6.5 Å in the Y149A^3.33^ and M2R simulations, reflecting a slightly increased instability of tiotropium, although we did not observe any unbinding event within this time frame for any of the systems (Figure S2). In M3R, a salt bridge between K523^7.32^ and E220^ECL2^ contributes to the stabilization of ECL2 (Figures 2B and S3). During the unbiased simulations, this salt bridge was observed to form and dissociate repeatedly over the 500 ns time frame (Figure S4), consistent with findings from previous MD simulations reported in the literature.^10^ In M2R, the lysine is replaced by N419^7.32^ and the stabilization given by the salt-bridge interaction is absent and likely contributes to the lower tiotropium RT.

Given that no unbinding event could be observed in our unbiased simulations, we performed preliminary ABMD simulations to tune the force constant applied during simulations, leading to complete solvation of the ligand. We defined the CV as the component of the distance between the centers of mass (COM) of tiotropium and the residues at the floor of the binding site normal to the plane of the membrane (Figure S5). With this setup, we assumed that the ligand unbinding route was pointing exclusively toward the solvent and alternative pathways toward the membrane bilayer were excluded. The ligand was considered fully dissociated when surrounded by only solvent molecules or ions within 6 Å, i.e., approximately two solvation shells. Based on the best resolution observed between slowest and fastest systems, we set a force constant value of 0.005 kcal·mol^–1^·Å^–4^ to systematically perform our simulations on 12 M3R systems with binding site mutations. The unbinding times obtained were then compared to the experimental dissociation rates through a linear regression analysis (Figure S6). The results showed that the method was able to moderately reconstruct the ranking of dissociation times (Spearman ρ 0.59). However, this relatively satisfactory result is particularly challenging, considering the extensive simulation time required for complete unbinding. Systems with longer RTs required over half a microsecond to reach the ABMD end point, totaling approximately 400 days of wall-clock time on a single NVIDIA GeForce RTX 3060 Ti GPU for running simulations on all mutants. Notably, the approach managed to approximately discriminate between two groups: one encompassing WT M3R and the mutants displaying longer RTs (p*k*_off_ > 1.5) and another including those mutants that caused a significant drop in RT (p*k*_off_ < 1.5). Considering the median computed RT (148.9 ns) as a threshold to classify mutants displaying slow or fast unbinding kinetics, a moderate performance in the classification of the mutants could be obtained (accuracy: 0.75; Matthews’ correlation coefficient: 0.51). For a pair of systems, namely, Y149A^3.33^ and W504A^6.48^, the unbinding time was overestimated, resulting in reduced correlation with the experimental residence time.

### Application of PCV-ABMD to the Study of Tiotropium Unbinding Kinetics

The distance-based ABMD approach presents inherent limitations related to the chosen CV. Indeed, distance projections might suffer from intrinsic degeneracy when describing the unbinding motion, as they offer an oversimplified representation of the system, leading to a loss of resolution and an inability to accurately describe transition or intermediate states along the exit pathway. For this reason, we decided to use PCVs^40^ that could account for multiple coordinated degrees of freedom of the system at the same time. Starting from the initial unbinding trajectory of tiotropium from WT M3R, we generated a frameset consisting of 20 states. The path consisted of an initial state, corresponding to tiotropium bound to the orthosteric site, and intermediate states leading to an end state with tiotropium in the vestibular site (Figure 3). Moreover, a harmonic potential restricting the access to orthogonal degrees of freedom was set in place and calibrated on the data set of preliminary distance-based ABMD simulations to allow potential variations from the initial guess path.

**Figure 3:**
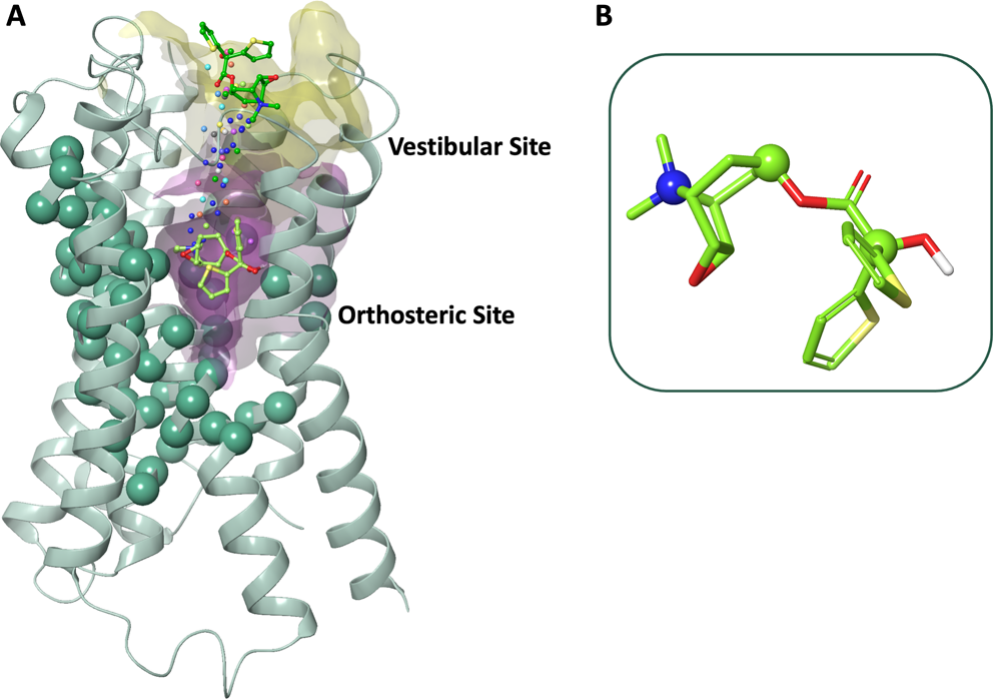
(A) Schematic representation of the atoms that are included in the definition of PCVs. The path nodes are represented by the tiotropium atom selection pictured as small spheres. The full heavy atom models of tiotropium in the first and last state of the path are represented as lime and light green ball-and-sticks, respectively. The protein C_α_ atom selection present in the path as alignment selection is represented as van der Waals spheres. Surface representations of the orthosteric and vestibular sites are depicted in purple and yellow, respectively. (B) 3D representation of tiotropium with light green sticks with highlights on the atoms included in the selection defining PCVs, represented as spheres.

We choose to only simulate the transition to the vestibule since the crossing of the first energetic barrier is the main hindering factor in the unbinding event, and it is likely responsible for tiotropium slow dissociation kinetics. The translocation was considered complete when the distance between the tiotropium quaternary nitrogen atom and the D148^3.32^ γ-carbon atom reached 12 Å. After comparing it with the S collective variable (Figure S7), this geometric criterion proved efficient in representing the translocation event. Specifically, once this cutoff was exceeded, the final states of the path were sampled, and tiotropium was found residing in the vestibule. Retrospectively, we analyzed the distance-based ABMD simulations showing how the translocation and unbinding events happened nearly simultaneously for all of the mutants (Figure S8). After an initial tuning, we set a harmonic force constant of 0.05 kcal·mol^–1^. Using the PCV-ABMD approach, the calculated mean times of translocation showed an improved correlation against the experimental p*k*_off_ values (Tables 1 and S1). Moreover, the method allowed for the application of a greater force constant, significantly decreasing the calculation times (182 GPU days) while enabling a good resolution in the results.

**Table 1:** Spearman and Pearson Coefficients at Different Cutoffs of the Applied Criteria to Assess Ligand Translocation to the Vestibular Site

	TIO_N-D148_Cγ distance (Å)			ligand RMSD (Å)			COM distance (Å)			% SASA			
	6	9	12	6	10	14	5	10	15	10	20	30	40
Spearman ρ	0.64	0.62	0.72	0.59	0.66	0.72	0.59	0.65	0.34	0.79	0.59	0.74	0.64
Pearson *r*	0.64	0.65	0.69	0.68	0.68	0.67	0.67	0.67	0.29	0.71	0.68	0.66	0.66

The obtained dispersion of the data points showed that the method was efficient at ranking those systems presenting mutations in the aromatic cage (Figure 2A), usually referred to as the “lid”, as well as I204A^4.61^, which presents the mutation on the egress route. The residence times of V156A^3.40^ and W504A^6.48^ were overestimated; conversely, F240A^5.47^ and Y534A^7.43^ were underestimated. The mutations in these systems are all located at the bottom of the orthosteric site, which suggests that some kind of effect involving the rearrangement of the cavity may have been overlooked in the initial stages of the simulations or presents orthogonal degrees of freedom not considered by PCVs.

To assess whether the number of replicas generated was sufficiently representative of a wider population, we performed a comparison of the mean ranking correlations obtained from the bootstrapped data set of the translocation times at different sample sizes (Figure S9). Indeed, we demonstrated that 15–20 replicas were adequate to obtain a significant representation of a wider population of events.

### Comparison among Multiple Criteria to Assess the Ranking of Tiotropium Residence Times in M3R Mutants

We analyzed the generated trajectories to estimate residence times by applying various criteria to define the translocation event from the orthosteric site to the vestibule. Each criterion was evaluated across a range of cutoff values. The assessment revealed that the use of geometric parameters was more efficient when applied to the later stages of the transition event (Table 1). As shown in Figures S10 and 4, the distance between the tiotropium quaternary nitrogen atom and the D148^3.32^ γ-carbon atom allowed a better estimation of the translocation times when the distance cutoff was set at 12 Å. The rank correlation as well as the strength of the linear correlation decreased when cut-offs at 9 and 6 Å were applied (Figure S10). A similar rank correlation was obtained using the root-mean-square deviation (RMSD) of the ligand with respect to the starting pose (Figure S11). The Spearman ρ was 0.59, 0.65, and 0.72 at RMSD cutoffs of 6, 10, and 14 Å, respectively. Conversely, we did not observe relevant changes in Pearson *r*. A slightly different trend was obtained by using the distance between the center of mass of the ligand and that of the binding site residues to define unbinding. In this case, the best correlation was observed using an intermediate cutoff of 10 Å, while values of the distance of 5 and 15 Å provided less accurate results (Figure S12). Similarly, the regression Pearson *r* was better at 5 and 10 Å but decreased drastically at a 15 Å cutoff.

**Figure 4:**
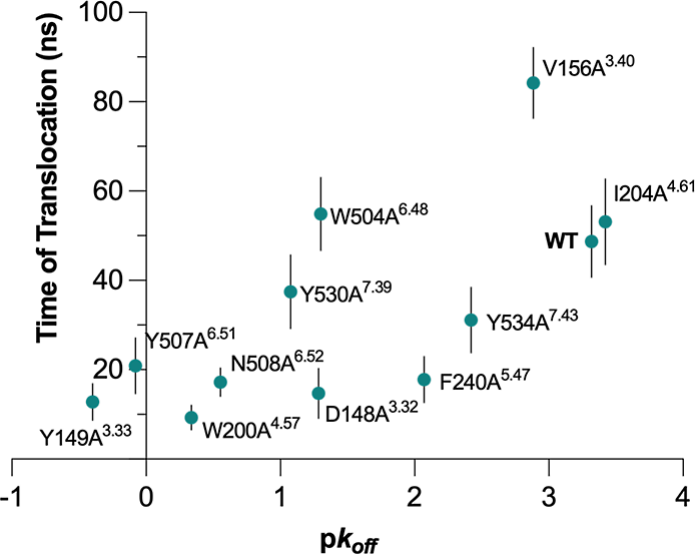
Plot of the experimental dissociation rates (p*k*_off_) versus calculated mean residence times (Spearman ρ = 0.72, Pearson *r* = 0.69). The error bars represent the standard error of the mean of 20 replicas.

Interestingly, using the fraction of solvent-accessible surface area (SASA) of the ligand as an unbinding criterion enabled discrimination among mutants based on events occurring at the very onset of the unbinding process (Figure S13). The strongest rank correlation and linear regression were achieved at a 10% cutoff, suggesting that solvation plays a critical role in the initial stages of unbinding (Table 1).

We also applied a method based on protein–ligand interaction fingerprints (IFP) developed by Kokh et al.^62^ To determine unbinding times, we monitored the variation in Tanimoto distance between instantaneous IFPs and the initial IFPs along the trajectory, testing different cutoffs. The unbinding event was defined as the point where the Tanimoto distance exceeded each threshold. Comparing the resulting rank correlation coefficients and linear regression strength (Figure S14) revealed the best agreement with experimental rates at a cutoff of 0.3. Similar to the case when % SASA was the monitored criterion, this cutoff value for Tanimoto distance suggests that early events in the unbinding process are more predictive of the overall RT of the ligand across different systems (Figure 5). These initial stages, likely involving ligand solvation and the weakening of interactions with binding site residues, may play a critical role in determining unbinding kinetics.

**Figure 5:**
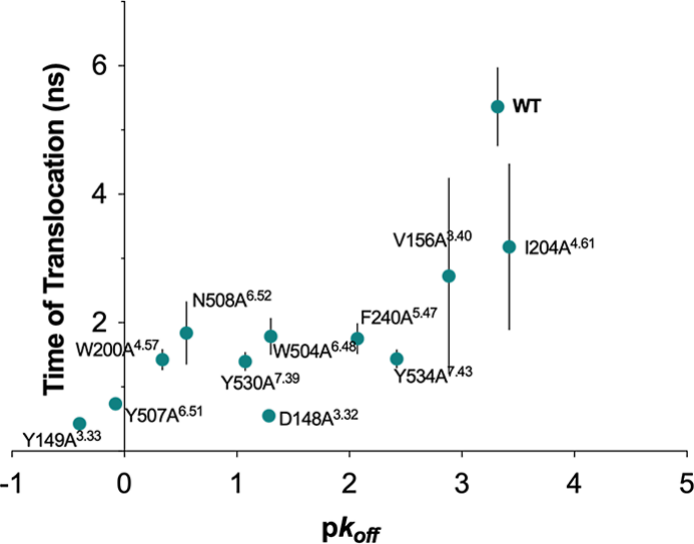
Plot of the experimental dissociation rates (p*k*_off_) versus calculated mean residence times estimated with a Tanimoto distance cutoff of 0.3 (Spearman ρ = 0.79, Pearson *r* = 0.78). The error bars represent the standard error of the mean of 20 replicas.

### Assessment of the PCV-ABMD Simulations for Ligand Unbinding and Kinetic Selectivity

To assess the linear regression model for M3R ligand unbinding kinetics, we evaluated additional systems other than the WT and the 11 orthosteric mutants. These systems included a mutant of the extracellular vestibule (K523A^7.32^) as well as two tiotropium analogues belonging to Tautermann’s data set. We also considered tiotropium unbinding kinetics at M2R. From our regression model, we discarded the two mutations characterized by the highest residual (i.e., mutants V156A^3.43^ and W504A^6.48^ with an increase of 34 and 24 ns in the simulated residence time, respectively, Figure 4), resulting in a total of ten data points.

We estimated the residence time of tiotropium at the K523A^7.32^ mutant, given the importance of this residue in the stabilization of the ECL2 in WT-M3R. The simulated mean time of translocation (36.3 ± 6.7 ns) was in good agreement with the prediction inferred by the linear correlation for the orthosteric mutants (41.2 ns).

Furthermore, we tested the protocol by simulating the translocation times of CPD9 (*k*_off_ = 2.06 × 10^–2^ min^–1^) and CPD1 (*k*_off_ = 2.57 × 10^–1^ min^–1^) (Figure 6), whose dissociation rates are reported in Tautermann’s data set. The predicted stability within the WT M3R binding site was compared with their parent compound. Indeed, these ligands have a decreased residence time since some of the key interactions are progressively loosened: the CPD9 interaction with N508^6.52^ is weakened due to the loss of the α-hydroxyl substituent, while the CPD1 half-life is strongly affected by the removal of a thiophene group. The ligands’ ranking was correctly predicted and matched with the mutant’s regression as well (Figure 7A), giving a good quantitative agreement with experimental results.

**Figure 6:**
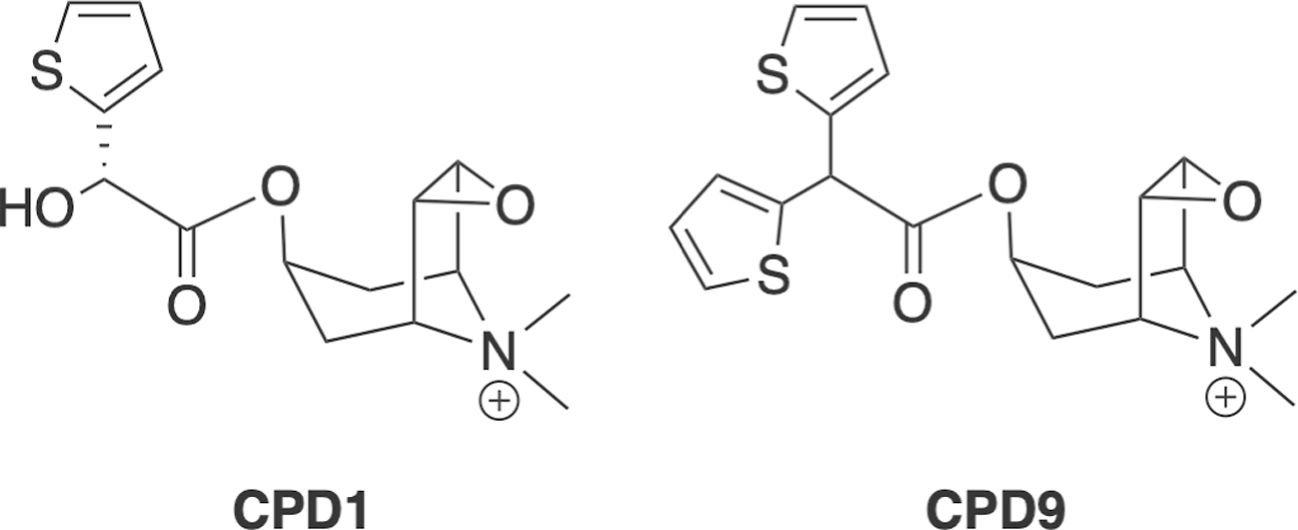
Tiotropium analogues endowed with medium and short residence time with respect to the parent compound.

**Figure 7:**
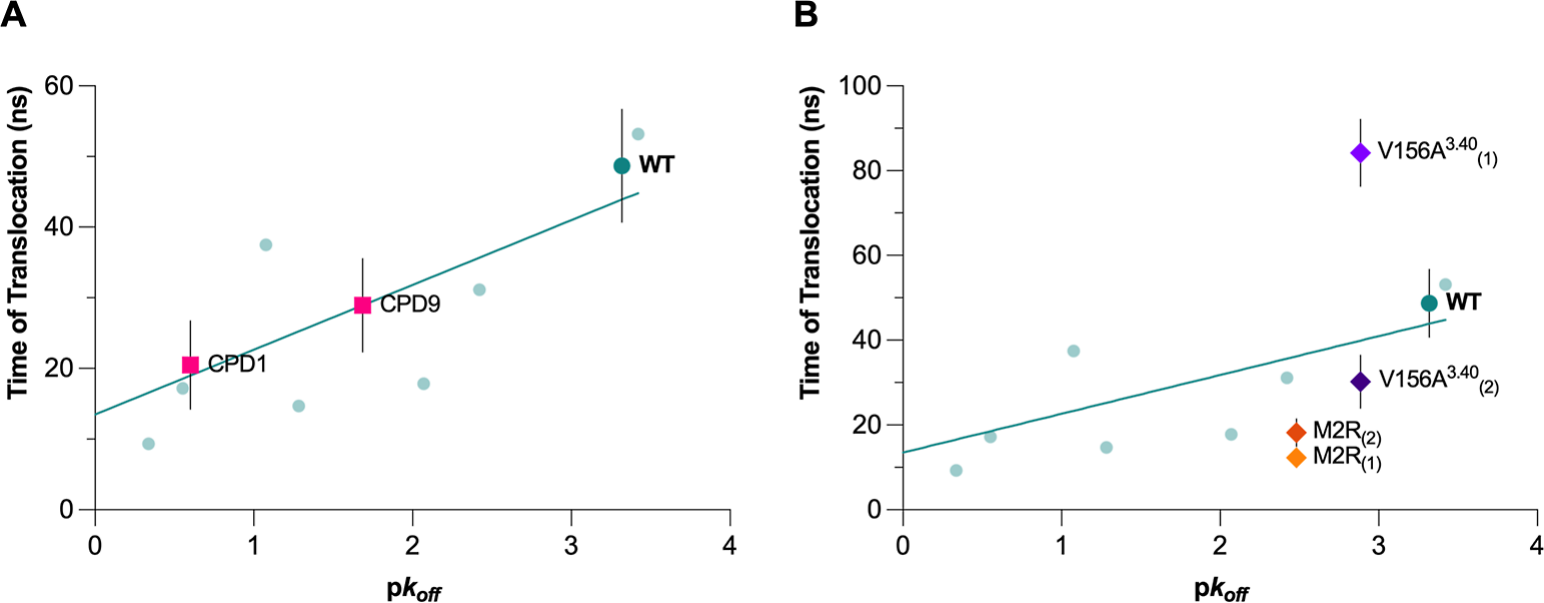
Evaluation of the PCV-ABMD protocol comparing the regression line of the orthosteric mutants with tiotropium analogues and highlighting the impact of system-dependent path optimization. The regression line was calculated using the mean residence time of the mutants set (with data points represented as pale green dots Figure 4), discarding the two major outliers (*R* ^2^ = 0.65). (A) Plot of the experimental dissociation rates (p*k*_off_) versus calculated mean residence times of tiotropium and the congeneric compounds CPD1 and CPD9. The residence times were calculated as the time when the distance between the tiotropium quaternary nitrogen atom and the D148 γ-carbon atom reached 12 Å. The error bars represent the standard error of the mean of 20 replicas. (B) Evaluation of PCV reparameterization for V156A^3.40^ and M2R. The data points (2) depicted with darker markers show the effect of the independent path optimizations.

The same protocol was applied to M2R to investigate the molecular determinants as the basis of the lower tiotropium RT. M2R was ranked correctly compared to M3R, although the mean translocation time (12.3 ± 2.0 ns) was underestimated with respect to the mutants’ regression, by which we had predicted a translocation time of 36.4 ns (Figure 7B). Therefore, the protocol might be accurate to rank mutants and ligands just at the M3R, since PCVs optimization could be system-dependent in regard to the estimation of the residence time.

To assess the reliability of the PCV-ABMD protocol, the frameset used as the guess path for tiotropium unbinding was re-parameterized for M2R and V146^3.40^A, to verify any eventual change in a different system and in the worst outlier among the orthosteric mutants (Figure 7B). Notably, for the V146^3.40^A mutant, the number of replicas in which the ligand remained trapped in the binding site decreased significantly, dropping from 16 to just two, thereby highlighting the importance of PCV parameterization in facilitating initial ligand unbinding events. In the case of M2R, such improvement was only marginal.

As already mentioned, a main difference between the two receptor subtypes resides in a dissimilar conformational freedom of the ECL2.^18^ Indeed, the flexibility of the loop during our unbinding simulations was greater at the M2R (Figure 8), in agreement with unbiased simulations (Figure S3) and in line with previous results,^10,18^ eventually allowing for an easier tiotropium egress in the vestibular site.

**Figure 8:**
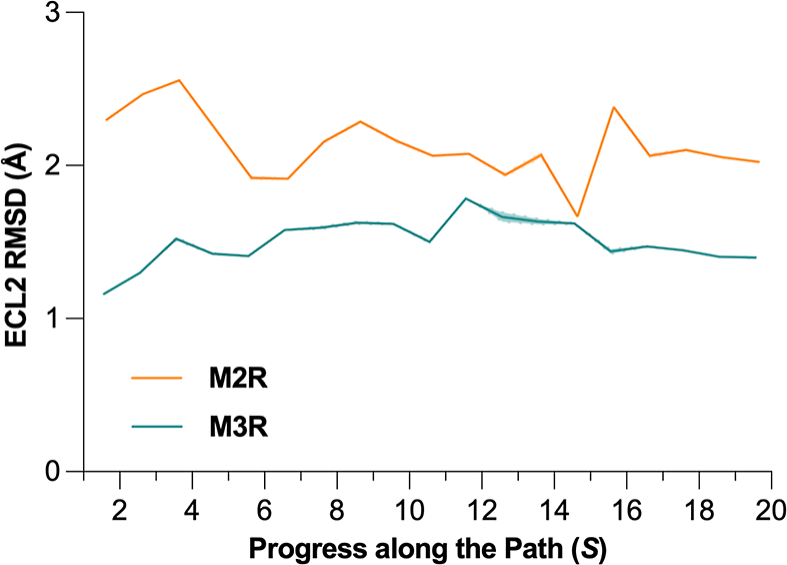
Conformational flexibility of the ECL2 in M3R and M2R (residues 212–223 and 167–184, respectively), monitored over binned cumulative distributions over the PCV representing the progress along the path for the translocation from the orthosteric site ( S < 4) to the extracellular vestibule ( S < 16).

### Dissecting the Contribution of Mutations in the Ligand Unbinding Pathway

To rationalize the impact of each mutation on the RT at the structural level, we performed a clustering analysis based on the contacts between the ligand and the binding site, according to the procedure described by Kokh et al.^36,62^ This analysis illustrated differences in the translocation of tiotropium among key systems affecting binding kinetics. Beyond WT-M3R and M2R, we also considered the D148A^3.32^ and N508A^6.52^ mutants to dissect the role of the two main polar interactions of tiotropium with the binding site. Last, we also considered Y149A^3.33^, the mutant with the shortest residence time for tiotropium. The goal of our analysis was to understand the main interactions established by tiotropium while translocating from within the orthosteric site toward the vestibule. As the ligand progresses along the exit route, it encounters a sequence of metastable states along the channel, whose stability may influence ligand RT. Each mutation can alter both the number and the characteristics of these metastable states. Thus, we analyzed frames from the production runs, extracted at an interval of 100 ps, from the beginning of the trajectory until a ligand RMSD cutoff of 14 Å relative to the starting structure was reached. Furthermore, we included an additional 5 ns beyond this point. This ensures that each trajectory describes the complete translocation of tiotropium to the vestibule while allowing for the sampling of the protein–ligand contacts once the ligand reaches this region.

In the case of WT-M3R, the clusters in the bound state at the orthosteric site appear to be more populated, reflecting the extended residence of the ligand at this stage (Figure 9A). These states are characterized by a ΔCOM, i.e., the distance between the instantaneous and initial positions of ligand COM, lower than 2 Å. The main detected interactions correspond to those revealed in the crystal structure, including the “snap-lock” hydrogen bonds with N508^6.52^. Clusters associated with an intermediate state of the ligand egression (ΔCOM 5–6 Å) are characterized by the loss of the hydrogen bonds with N508^6.52^. This bond is replaced by an interaction of the same type with Y507^6.51^. This residue contacts the ester portion of tiotropium when the ligand rotates along the unbinding channel. Of the interactions formed in the starting ligand conformation, aromatic interactions with F222^ECL2^ and W526^7.35^ are the only ones consistently maintained, remaining present in the final cluster when the ligand has fully translocated into the vestibule (Figure 9B).

**Figure 9:**
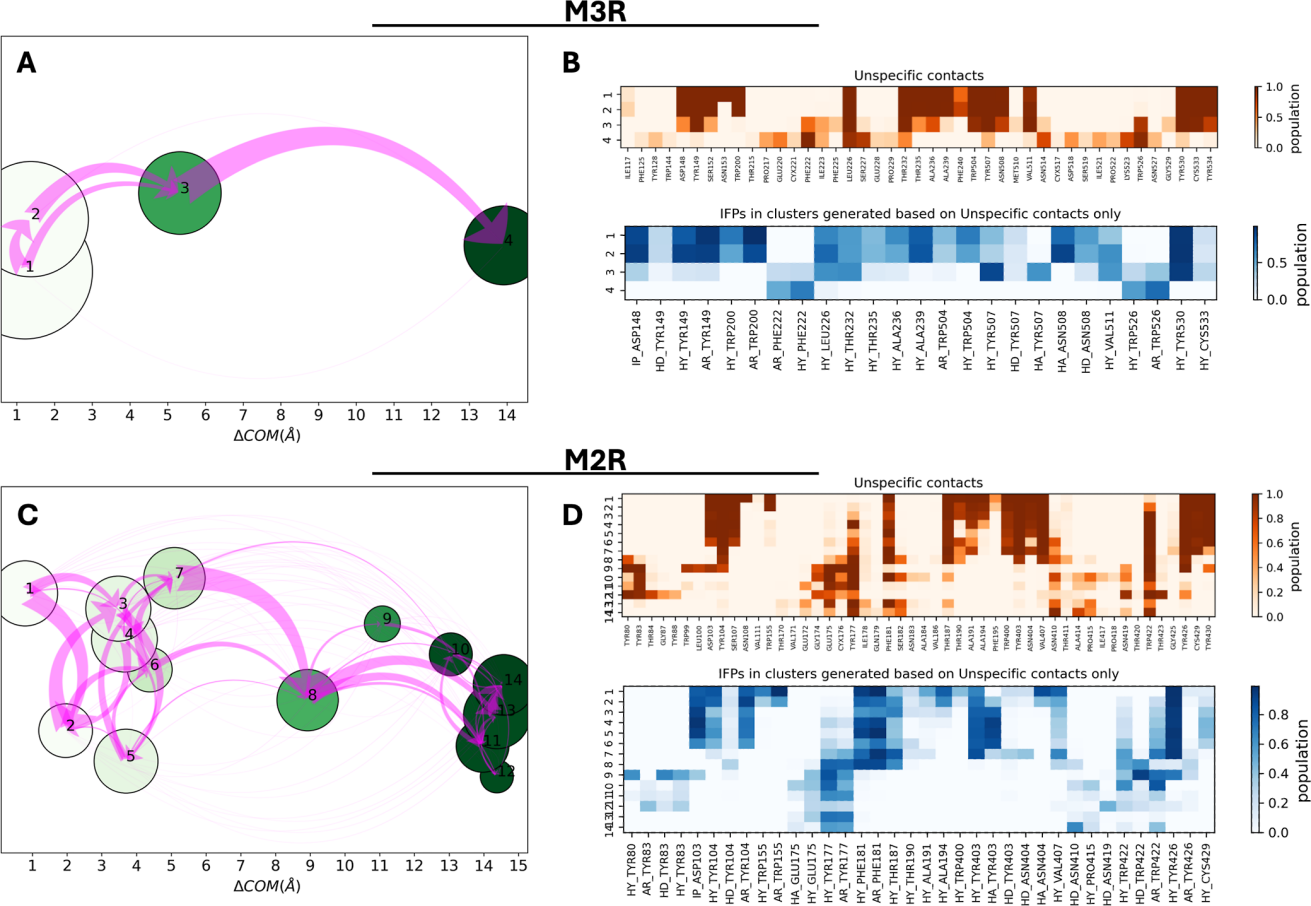
Schematic representation of the trajectory clustering analysis of WT-M3R (A,B) and M2R (C,D). Left: Clusters are shown by nodes positioned along the *x*-axis based on their ligand mean ΔCOM and the size is proportional to the cluster population. The green color of the nodes is darkened according to the increased averaged ligand RMSD in the cluster from the starting structure. The magenta arrows indicate the total net flow between two nodes. Right: IFP composition of each cluster. Nonspecific protein–ligand contacts and IFPs are represented in orange and blue maps, respectively.

The clustering of M2R shows significant differences with respect to that of WT-M3R, starting from the number of clusters (Figure 9C). Notably, only two small clusters corresponding to the bound state are detected at ΔCOM < 2 Å, suggesting how tiotropium is only marginally stabilized for short simulation times. In frames from clusters 3 to 7, those ranging in ΔCOM between 2 and 5 Å, the main initial interactions are progressively lost. In particular, the snap lock with N404^6.52^, already weakened in the early transition between clusters 1 and 2, completely disappears; at the same time, a hydrogen bond with Y403^6.51^ is formed. The ionic interaction with D103^3.32^ persists in the first 6 clusters and is abruptly lost between clusters 6 and 7. Aromatic interactions with Y177^ECL2^ and W422^7.35^ progressively intensify in the intermediate states denoting a more continuous transition compared to M3R (Figure 9D).

The analysis of the D148A^3.32^ system revealed a small cluster at a lower ΔCOM compatible with the bound state (Figure S15). Abrupt transitions to clusters 2, 3, and 4 occur due to the absence of Coulombic interactions caused by the mutation, which provides greater freedom for the polar head of tiotropium to move. The snap-lock interactions with N508^6.52^ are only partially detected in cluster 1, probably due to the transient nature of this state. The clusters that follow in the IFP map describe a scenario similar to the one associated with the last two clusters of the WT system. However, in D148A^3.32^, this last transition happens more gradually (Figure S15A,B). Similarly, in the Y149A^3.33^ system, the stability of the bound pose, expressed by the number of frames populating cluster 1, is reduced with respect to WT-M3. However, the ionic interaction with D148^3.32^ is maintained until the intermediate state in cluster 4 at a ΔCOM of 8–9 Å. No hydrogen-bond interaction with N508^6.52^ is detected at any stage. The presence of interactions with F222^ECL2^ in all early clusters denotes a propensity, permitted by the lack of the aromatic ring of Y149^3.33^, of the thiophene rings of the ligand to move toward the vestibule (Figure S15C,D). In the case of the N508A^6.52^ system, the two clusters associated with the bound state show similar IFPs, differing only for the formation of a hydrogen bond interaction with Y507^6.51^. The ligand loses the aromatic interactions with W200^4.57^ and W504^6.48^ in the state denoted by cluster 3 and gains aromatic interactions with F222^ECL2^ and W526^7.35^. This change is consistent with the movement of the thiophene rings toward the exit channel. These interactions persist for the rest of the trajectory (Figure S15E,F).

## Discussion

The orthosteric site of muscarinic receptors is highly conserved, posing a challenge for the design of selective ligands. Achieving greater subtype selectivity for M3R over M2R, thus minimizing side effects, could potentially be accomplished by targeting subtle differences in the orthosteric site. This can be achieved by introducing substituents optimized to occupy a specific subpocket, thereby enhancing selectivity.^63^ However, a more common strategy for achieving selectivity across muscarinic subtypes is to consider RT as a critical parameter.^64^ This approach involves rationalizing subtle structure–kinetic relationships and considering the entire unbinding process. In this light, developing a reliable computational protocol capable of predicting the impact of mutations on RT would be highly valuable. Here, we attempted to correlate the experimental dissociation rates reported by Tautermann et al.^10^ with computed unbinding times. We relied on a PCV-ABMD protocol to simulate the translocation of ligand from the orthosteric site to the vestibular site. Indeed, the unbinding of M3R antagonists involves two primary energetic barriers: the first is associated with ligand translocation through the tyrosine cage from the orthosteric site to the vestibule and the second corresponds to the final exit from the vestibule into the bulk solvent. The first barrier is energetically higher and it has been extensively identified as the main limiting step of the unbinding process.^10,18,33,39^ Hence, in analogy with previous work,^39^ we attempted to obtain an acceptable approximation of the experimental results by just simulating the translocation across the main barrier as this is the major event impacting the unbinding rates. Indeed, when considering the first set of simulations, the time required for the translocation from the orthosteric site to the extracellular vestibule and that required for a complete unbinding fully aligned (Figure S8).

We proposed the application of PCVs which reportedly allow for an improved sampling of low-energy transition states compared to simpler CVs, such as distances or RMSD.^40^ In our study, we obtained rankings of dissociation rates by applying an agile and generalized approach, which provided consistent results in relatively short simulation times. Compared to the time-consuming results based on the distance CV obtained in an earlier phase of the work, with PCVs, we were able to apply a higher force constant and observe the translocation event within 100 ns for most of the tested systems. Furthermore, our bootstrapping analysis demonstrated that the accuracy of our predictions could be achieved with fewer than the 20 replicas performed (Figure S9). Therefore, our protocol effectively balances thorough exploration of molecular flexibility with efficiency, delivering results within a time frame suitable for fast-paced drug discovery programs.^26^ Indeed, in terms of simulation times, PCV-ABMD performed similarly to τRAMD, which, to our knowledge, is one of the most efficient among the reported methods for the analysis of unbinding events.^36^

As shown in Figure 4, PCV-ABMD simulations, with a maximum runtime of 100 ns, achieved a good ranking correlation with the experimental dissociation rates (Table S1). However, the presence of several outliers negatively skews our results. On the one hand, the RT of V156A^3.40^ and W504A^6.48^ was significantly overestimated. Interestingly, both residues are located at the bottom of the binding cavity. The behavior of tiotropium within these systems might be inaccurately estimated due to potential differences in its translocation path overlooked by our defined PCV. On the other hand, F240A^5.47^ and Y534A^7.43^ present a minor yet evident underestimation of the RT. This deviation may be similarly correlated to their position, as these residues also lie at the bottom of the binding site. Comparing the results with the initial set of simulations (Figure S6) further reinforces the idea that the discrepancy arises from the imposed CV. Notably, path reparameterization in the case of the V156A^3.40^ mutant qualitatively improved the results, suggesting the importance of a system-dependent selection, as recently shown in unbinding metadynamics relying on PCVs generated from initial system-dependent τ-RAMD.^65^ Moreover, while we did not observe the higher heterogeneity seen, for example, during the unbinding simulations of iperoxo at the M2R,^31,36^ it cannot be discarded that multiple unbinding pathways may exist. However, consistently applying path re-optimization would require longer initial runs, increasing the workload and undermining the goal of a time-efficient approach, with potentially limited gains in accuracy. Overall, we conclude that the path selection strategy should be carefully assessed based on the knowledge of the specific case study.

Another possible reason our protocol struggles to accurately describe the behavior of mutants located at the floor of the binding site is that solvation is not explicitly taken into consideration in the design of CVs describing the unbinding pathway. Solvation is crucial in protein–ligand dynamics, and several authors have explored the involvement of individual water molecules within protein cavities.^66,67^ In our case, the time required for a proper hydration of the receptor may be longer than that employed to equilibrate the systems, or the presence of the ligand might prevent a proper rearrangement of water molecules in the buried portions of the receptor. Moreover, the stability of the tyrosine cage might contribute toward hampering the ingress of water molecules.^33^ The crucial role of solvent molecules has also been previously recognized for tiotropium unbinding kinetics,^39^ since the presence of highly stable water molecules within the binding site, potentially stabilizing the transition states, could improve the description of the unbinding process.

Our extensive exploration of multiple criteria to define unbinding has shown that geometric parameters, i.e., RMSD, the distance between the ligand’s center of mass and the center of mass of selected orthosteric pocket residues, or the distance between two selected atoms, directly linked to the ligand translocation (Figures 4 and S10–S12) are more effective at determining the correct ranking when the ligand gets closer to the vestibular site. These geometric parameters are not independent and typically yield similar rankings with only minor differences in performance metrics. However, these variations may be system-dependent and influenced by the chosen collective variables, highlighting the value of comparing criteria in future applications. The analysis of water solvation in terms of % SASA revealed the best correlation at lower cutoffs (Table 1), once again hinting at the importance of solvation in the earliest stages of the unbinding event. Other than extensive preliminary simulations to establish the correct water networks in buried sites before biased simulations,^66,67^ we speculate that alternative strategies to improve the discrimination among the mutants could include employing a collective variable explicitly describing solvation^49^ or a hydration term for the binding pocket in the PCVs. These options will be explored in future work. Solvation effects are likely crucial in describing the unbinding process, particularly within buried binding sites, and the omission of such degrees of freedom may potentially explain the occurrence of instances in which the ligand remains artificially trapped within the binding site. Advancements in data-driven collective variables show that water coordination patterns assist in the modeling of ligand unbinding kinetics^49^ and in finding reactive conformations for enzyme catalysis.^68^

Our estimation of dissociation rates based on IFPs successfully captured subtle structural differences in ligand–protein interactions during the early stages of translocation, which were sufficient to correctly rank the mutants in terms of residence times. In other words, a fairly accurate correlation could already be recovered at short simulation times, with tiotropium still within the orthosteric binding site (Figures 5 and S14), highlighting the importance of key features such as the hydrogen bond that the α-hydroxy substituent forms with N508^6.52^. Notably, this interaction was generally the first to break and highly contributes to tiotropium tight binding and high residence time. These results obtained in IFP space are in conceptual agreement with the work of D’Arrigo et al.,^59^ in which distance and interaction-based metrics were compared in terms of efficiency. As in our case, their outcome revealed an improvement in recovering experimental rankings when interaction-based criteria were applied. Furthermore, an increased accuracy in the estimation of systems with faster off-rates could be observed. Finally, both studies suggest that this type of framework could be particularly suitable for studying the effect of mutations on RT.

Despite the major contribution of the first barrier to determining dissociation rates, the importance of ligand binding at the M3R vestibular site should also be considered. Unbiased^18,69^ and accelerated MD^70^ simulations have previously identified a metastable state of tiotropium in the vestibule. Indeed, the pharmacological relevance of tiotropium binding to the extracellular site is also confirmed by competition studies.^69^ Nevertheless, the contribution of tiotropium binding to the vestibule still has a limited impact on the residence time,^10^ and a good correlation with experimental data was already achieved by just simulating the translocation event.

The model obtained by regression studying the orthosteric mutants was also validated, applying it to tiotropium analogues with varying dissociation rates. Additionally, mutant K523^7.32^ from the vestibule was also investigated for its effect on the residence time (Figure 6). As already mentioned, lysine can establish a salt bridge with a glutamate belonging to ECL2. However, this interaction stabilizing the architecture of the vestibule for M3R might not be enough to rationalize kinetic selectivity, since the salt bridge is continuously formed and broken during simulations (Figure S2). Tautermann and colleagues postulated that this residue could represent an electrostatic barrier repelling the tiotropium quaternary nitrogen atom.^10^ However, in our simulations, the ammonium group of the *N*-alkyltropane moiety was oftentimes located on the opposite side with respect to K523^7.32^. This might be due to the intrinsic configuration of the PCV frameset. Nevertheless, by the end of the simulations, none of the interactions with the residues of the extracellular vestibule were directly discouraged by the application of PCVs. Indeed, the repulsive potential over the Z CV, representing the distance from the path, was calibrated considering the entire data set of preliminary simulations. Still, the way PCVs are defined may influence the orientation of the charged ammonium group during translocation. In PCV-ABMD simulations, the thiophene rings represent the first portions of tiotropium to exit from the orthosteric site, in agreement with what has already been reported in metadynamics simulations.^33^ However, even in our preliminary simulations, the ligand orientation with the charged ammonium group pointing backward to the orthosteric site was rarely observed, and only in some systems, contrary to that reported by Buigues et al.^39^ These observations were also confirmed using our clustering procedure based on the IFP metrics. The analysis furthermore elucidated the presence of possible metastable states along the unbinding route (Figures 9 and S15 and S16). In particular, these states could be observed with an increased frequency in mutants endowed with faster unbinding kinetics, as they likely depend on the destabilization of receptor–ligand interactions at the bound state. When comparing M3R and M2R, metastable states are probably linked to the ECL2 conformational flexibility which is increased in M2R (Figures 8 and S3), coherently with previous computational studies.^10,18^ The enhanced ECL2 flexibility at the M2R allows for the presence of alternative metastable states that are thoroughly sampled thanks to the application of PCVs in our unbinding simulations.

## Conclusions

Rare events, such as the unbinding of M3 receptor antagonists, happen on a time scale that is not practically accessible to unbiased MD simulations.^71^ Using our PCV-ABMD method, we obtained a stronger correlation between the simulated times necessary for the translocation to the extracellular vestibule and the experimental dissociation rates of M3R mutants. Our observations were consistent with previous studies^10,18,33,39^ and emphasized the weight of the first energetic barrier on the unbinding process of muscarine receptor antagonists. While out-of-equilibrium dynamics simulations can be used to retrieve correct ligand binding kinetic rates,^72^ our approach is more focused on the ranking of systems with a pharmaceutical interest, as ABMD previously showed potential in correctly ranking congeneric compounds.^50^ Hence, despite our approximations, we were able to assess differences in the unbinding trajectories, rationalizing the impact of mutations and the kinetic selectivity of tiotropium at the M3 and M2 receptor subtypes. Indeed, an extensive analysis of protein–ligand contacts afforded the identification of frequently accessed metastable states, potentially rationalizing the lower RTs of M3R mutants and the faster dissociation kinetics of M2R.

Our protocol utilized preliminary simulations to construct initial guess paths for unbinding trajectories performed through ABMD simulations. This approach is suitable for systems where simple geometric variables fall short of capturing complex unbinding mechanisms such as GPCRs with deeply buried binding sites. This strategy can be applied to understand structure–kinetics relationships for a series of congeneric compounds, under the assumption that a common unbinding mechanism is shared. To this end, the combination of ABMD with the PCVs can be seen as a relatively affordable and precise protocol aimed at ranking compounds based on kinetic dissociation rates during the ligand optimization stage of drug discovery while also recovering potentially interesting metastable states along the unbinding route.

## Methods

### System Preparation

Complexes of the rM3 and hM2 receptors (PDB codes 4DAJ^18^ and 3UON,^61^ respectively) were prepared with Schrodinger-Suite 2023-01 ^73^ and fusion constructs were removed. In the case of the rM3 receptor, chain A was selected, considering the highest ligand real-space correlation coefficient (RSCC). Moreover, amino acids V65^1.29^, I77^1.41^, S92^1.56^, A146^3.30^, F525^7.33^, and M557^8.56^, present in the human receptor, were reintroduced. For both M3R and M2R, intracellular loop 3 (ICL3) was not rebuilt and the termini were capped with neutral groups (acetyl and methyl-amide). D114/103^3.32^ was kept in the charged form, considering the inactive state of both receptors.^74^ The hydrogen-bonding network in the structure was then optimized at pH 7.4 and the resulting structure relaxed and minimized using the OPLS4 force field^75^ with heavy atoms constrained to a maximum RMSD of 0.3 Å from the initial structure. The Ramachandran plot of the minimized structure of hM3R showed that all backbone dihedral angles belong to permitted regions (Figure S17).

Complexes of hM3R with CPD1 and CPD9 reported by Tautermann et al.^10^ and the complex of hM2R with tiotropium (Figure S18) were obtained through docking calculations performed with Glide v10.3 ^76,77^ with standard precision mode. The docking receptor grid was centered on the bound ligand for both receptors, setting a bounding box of 10 Å^3^, and the calculations were run with default settings. The complexes retrieved from docking calculations were minimized using the OPLS4 force field^75^ with MacroModel v. 14.4 to an energy gradient of 0.01 kJ·mol^–1^·Å^–1^, considering the ligand and the residue side chains within 5 Å as flexible and freezing the position of the receptor backbone.

All systems were embedded in a POPC membrane bilayer of 80 Å^2^ using the CHARMM-GUI.^78^ Moreover, the bilayer was solvated in a TIP3P water box, considering a thickness of 22.5 Å on both sides of the membrane and an ionic concentration of 0.15 M NaCl. The system was parameterized using BiKiLifeSciences v. 1.3.5,^79^ employing Amber ff14SB^80^ for protein atoms, GAFF for ligands,^81^ Lipid14 for the membrane, and TIP3P as the water model.^82^ Ion parameters were assigned according to Joung and Cheatham.^83^ Charges for tiotropium and the other ligands were computed through RESP calculations^84^ with the HF/6–31G* level of theory and basis set using BiKiLifeSciences v. 1.3.5.^79^

### Molecular Dynamics Simulations

After equilibration (described in Supporting Information), unbiased simulations of every complex were performed with AMBER22 ^85^ for 100 ns to further equilibrate the systems for the unbinding simulations. However, for some of the systems (WT, Y149^3.33^, and hM2R), the unbiased production was prolonged until 500 ns. Temperature (303 K) and pressure (1 atm) were enforced with a Langevin thermostat^86^ and Berendsen barostat,^87^ using semi-isotropic pressure scaling. Bonds between hydrogen atoms and heavy atoms were constrained using the SHAKE algorithm^88^ to allow for a time step of 0.002 ps. Trajectory snapshots were recorded every 5000 steps. Full electrostatic and van der Waals interactions were computed within a cutoff of 9 Å, and long-range electrostatic interactions were treated using the Particle Mesh Ewald algorithm.^89^

### Adiabatic-Bias Molecular Dynamics (ABMD) Simulations

Adiabatic-bias MD, also known as ratcheted MD,^57^ is a free-energy method implemented in PLUMED^90^ which only exploits thermal fluctuations to induce the movement of the system along the chosen collective variable (CV). This is achieved by applying a biasing harmonic potential dependent on the exploration achieved with the sole thermal fluctuation of the system, in the form of

$$U\left(\rho \left(t\right)\right)=\{\begin{array}{l} \frac{k}{2}{\left(\rho \left(t\right)-\rho_{m}\left(t\right)\right)}^{2};\;\rho \left(t\right)>\rho_{m}\left(t\right)\\ 0;\;\rho \left(t\right)\leq \rho_{m}\left(t\right) \end{array}$$

where ρ_m_(*t*) is the minimum value of ρ­(*t*) explored during the simulation. Considering CV_0_ as the target value to be reached at the end of each of the replicas, and CV_*i*_ as the instantaneous position of the system, the two parameters ρ­(*t*) and ρ_m_(*t*) are defined as follows:

$$\rho \left(t\right)={\left({CV}_{i}-{CV}_{0}\right)}^{2}$$

$$\rho_{m}\left(t\right)=\min_{0\leq \tau \leq t}\left(\rho \left(t\right)\right)$$

With this technique, different approaches are reported depending on the used CV.

### Distance-Based Protocol

An initial set of simulations consisting of five replicas for each system considered the unbinding CV as the component normal to the plane of the membrane of the distance between the center of mass (COM) of tiotropium and residues at the floor of the binding site (Figure S5). The binding site COM was defined by the α carbon atoms of the following residues: S152^3.36^, N153^3.37^, V156^3.40^, A239^5.46^, and W504^6.48^. For each system, a force constant of 0.005 kcal·mol^–1^·Å^–4^ was applied to the CV until a final distance of 30 Å.

### Path-Based Protocol (PCV-ABMD)

An additional set of simulations (20 replicas for each system) is performed using path-collective variables (PCVs),^40^ two high-dimensional CVs defined as

$$S=\frac{\sum_{i=1}^{P}ie^{-\lambda {\left[(x-x_{i})\right]}^{2}}}{\sum_{i=1}^{P}e^{-\lambda {\left[(x-x_{i})\right]}^{2}}}$$

$$Z=-\lambda^{-1}\ln\sum_{i=1}^{P}e^{-\lambda {\left[(x-x_{i})\right]}^{2}}$$

which represent the progress of the system along a set of reference configurations *x*_*i*_ (between 0 and *P*, where *P* is the number of frames comprised in the frameset) and the distance of the position of the system (*x*) from the reference frameset, respectively. The distance matrices included in the definition of PCVs are measured in the mean-square deviation space, as implemented within the PATHMSD function of PLUMED.^90^ The smoothness of the two collective variables is regulated through a tuning parameter λ, which is set equal to 0.71 Å^–2^, according to the following rule of thumb:

$$\lambda =\frac{2.3P}{\sum_{i=1}^{P}{\left[(x-x_{i})\right]}^{2}}$$

Our frameset was defined in the form of 20 frames comprising a selection of receptor and ligand atoms (Figure 3). For the receptor, a list of α carbon atoms was made to align the ligand configurations. This selection was made considering a threshold of the root-mean-square fluctuation (RMSF) of 0.6 Å in a 500 ns plain MD simulation of the M3R–tiotropium complex (Figure S19). Instead, selected heavy atoms of tiotropium and the other ligands were included, roughly corresponding to the major axis of inertia (Figure 3B) (i.e., the ammonium quaternary nitrogen, the tropane carbon in position 7, and the α carbon of the ester chain) to consider ligand displacement.

A force constant of 0.05 kcal·mol^–1^ was applied to force the system to reach S = 20, which corresponds to a final state in which tiotropium resides in the vestibular site. The exploration of system positions with Z ≥10 Å^2^ was discouraged through the application of an upper wall consisting of a harmonic potential with a force constant of 1,000 kcal·mol^–1^·Å^–4^. This upper wall was calibrated by considering tiotropium unbinding in all preliminary simulations of M3 orthosteric mutants.

The protocols for the generation and optimization of the frameset are thoroughly reported in the Supporting Information. The frame selection was based on RMSD clustering of initial ABMD replicas, followed by steered MD simulations^91^ to optimize the definition of PCVs. The procedure was also applied for the V156A^3.40^ mutant and M2R.

### Unbinding Simulation Analysis

Several criteria for stopping the unbinding simulations were implemented using in-house VMD-Python scripts.^92^ The first criterion defined the unbinding event as when the ligand is solely surrounded by solvent molecules and ions within 6 Å (equivalent to two solvation shells). Instead, when considering the translocation to the vestibular site, we considered a distance of 12 Å between the carboxylate group of D114/103^3.32^ and the ligand quaternary nitrogen atom as the upper limit of the free-energy basin associated with the bound state.

We employed in-house VMD-Python-based scripts^92^ to perform the analyses of distances, RMSD and SASA. Analyses based on IFP-metrics^62^ are described in the Supporting Information.

## Supplementary Material



## Data Availability

Simulations were carried out with AMBER22 and AmberTools (https://ambermd.org/), which are distributed under license. PLUMED (https://www.plumed.org/) is an open-source plugin for MD to allow for enhanced sampling simulations and collective variable design. Schrödinger Suite (https://www.schrodinger.com) and Biki Life Sciences (http://www.bikitech.com/) were used to prepare and parameterize the systems and are distributed under license. Representative input data for PCV-ABMD simulations used during this study have been deposited to the public repository of the PLUMED consortium, PLUMED-NEST (plumID:25.002).

## Notes

Detailed information regarding analysis of MD and ABMD simulations, generation and optimization of PCVs, and clustering protocol based on IFP metrics (PDF)

## References

[1] Copeland R. A., Pompliano D. L., Meek T. D. (2006). Drug–Target Residence Time
and Its Implications for Lead Optimization. Nat. Rev. Drug Discovery.

[2] Swinney D. C. (2008). Applications
of Binding Kinetics to Drug Discovery: Translation of Binding Mechanisms
to Clinically Differentiated Therapeutic Responses. Pharm. Med..

[3] Copeland R. A. (2016). The Drug–Target
Residence Time Model: A 10-Year Retrospective. Nat. Rev. Drug Discovery.

[4] Borisov D., Veselovsky A. (2020). Ligand–Receptor Binding Kinetics in Drug Design. Biochem. (Moscow), Suppl. Ser..

[5] Paton W. D. M. (1961). A Theory
of Drug Action Based on the Rate of Drug-Receptor Combination. Proc. R. Soc. Lond. B Biol. Sci..

[6] Swinney D., Haubrich B., Liefde I., Vauquelin G. (2015). The Role of
Binding Kinetics in GPCR Drug Discovery. Curr.
Top. Med. Chem..

[7] Copeland R. A. (2010). The Dynamics
of Drug-Target Interactions: Drug-Target Residence Time and Its Impact
on Efficacy and Safety. Expert Opin. Drug Discovery.

[8] Guo D., Hillger J. M., Ijzerman A. P., Heitman L. H. (2014). Drug- Target Residence
Time–A Case for G Protein-Coupled Receptors. Med. Res. Rev..

[9] Pan A. C., Borhani D. W., Dror R. O., Shaw D. E. (2013). Molecular Determinants
of Drug–Receptor Binding Kinetics. Drug
Discovery Today.

[10] Tautermann C. S., Kiechle T., Seeliger D., Diehl S., Wex E., Banholzer R., Gantner F., Pieper M. P., Casarosa P. (2013). Molecular
Basis for the Long Duration of Action and Kinetic Selectivity of Tiotropium
for the Muscarinic M3 Receptor. J. Med. Chem..

[11] Casarosa P., Bouyssou T., Germeyer S., Schnapp A., Gantner F., Pieper M. (2009). Preclinical Evaluation
of Long-Acting Muscarinic Antagonists:
Comparison of Tiotropium and Investigational Drugs. J. Pharmacol. Exp. Ther..

[12] Disse B., Reichl R., Speck G., Traunecker W., Rominger K. L., Hammer R. (1993). Ba 679 BR, a Novel Long-Acting Anticholinergic
Bronchodilator. Life Sci..

[13] Disse B., Speck G. A., Rominger K. L., Witek T. J., Hammer R. (1999). Tiotropium (Spiriva): Mechanistical
Considerations
and Clinical Profile in Obstructive Lung Disease. Life Sci..

[14] Guo D., Dijksteel G. S., Van Duijl T., Heezen M., Heitman L. H., Ijzerman A. P. (2016). Equilibrium
and Kinetic Selectivity Profiling on the
Human Adenosine Receptors. Biochem. Pharmacol..

[15] Guo D., Heitman L. H., Ijzerman A. P. (2017). Kinetic
Aspects of the Interaction
between Ligand and G Protein-Coupled Receptor: The Case of the Adenosine
Receptors. Chem. Rev..

[16] Legros C., Devavry S., Caignard S., Tessier C., Delagrange P., Ouvry C., Boutin J. A., Nosjean O. (2014). Melatonin MT _1_ and MT _2_ Receptors
Display Different Molecular Pharmacologies
Only in the G -protein Coupled State. Br. J.
Pharmacol..

[17] Bedini A., Elisi G. M., Fanini F., Retini M., Scalvini L., Pasquini S., Contri C., Varani K., Spadoni G., Mor M., Vincenzi F., Rivara S. (2024). Binding and Unbinding of Potent Melatonin
Receptor Ligands: Mechanistic Simulations and Experimental Evidence. J. Pineal Res..

[18] Kruse A. C., Hu J., Pan A. C., Arlow D. H., Rosenbaum D. M., Rosemond E., Green H. F., Liu T., Chae P. S., Dror R. O., Shaw D. E., Weis W. I., Wess J., Kobilka B. K. (2012). Structure and Dynamics of the M3Muscarinic Acetylcholine
Receptor. Nature.

[19] Moulton B. C., Fryer A. D. (2011). Muscarinic Receptor Antagonists,
from Folklore to Pharmacology;
Finding Drugs That Actually Work in Asthma and COPD. Br. J. Pharmacol..

[20] Hughes A. D., Chen Y., Hegde S. S., Jasper J. R., Jaw-Tsai S., Lee T.-W., McNamara A., Pulido-Rios M. T., Steinfeld T., Mammen M. (2015). Discovery of (*R*)-1-(3-((2-Chloro-4-(((2-Hydroxy-2-(8-Hydroxy-2-Oxo-1,2-Dihydroquinolin-5-Yl)­Ethyl)­Amino)­Methyl)-5-Methoxyphenyl)­Amino)-3-Oxopropyl)­Piperidin-4-Yl
[1,1′-Biphenyl]-2-Ylcarbamate (TD-5959, GSK961081, Batefenterol):
First-in-Class Dual Pharmacology Multivalent Muscarinic Antagonist
and β _2_ Agonist (MABA) for the Treatment of Chronic
Obstructive Pulmonary Disease (COPD). J. Med.
Chem..

[21] Hulme E. C., Trevethick M. A. (2010). Ligand
Binding Assays at Equilibrium: Validation and
Interpretation. Br. J. Pharmacol..

[22] Motulsky H. J., Mahan L. C. (1984). The Kinetics of
Competitive Radioligand Binding Predicted
by the Law of Mass Action. Mol. Pharmacol..

[23] Lane J. R., May L. T., Parton R. G., Sexton P. M., Christopoulos A. (2017). A Kinetic
View of GPCR Allostery and Biased Agonism. Nat.
Chem. Biol..

[24] Wacker D., Wang S., McCorvy J. D., Betz R. M., Venkatakrishnan A. J., Levit A., Lansu K., Schools Z. L., Che T., Nichols D. E., Shoichet B. K., Dror R. O., Roth B. L. (2017). Crystal
Structure of an LSD-Bound Human Serotonin Receptor. Cell.

[25] Swinney D. C., Beavis P., Chuang K., Zheng Y., Lee I., Gee P., Deval J., Rotstein D. M., Dioszegi M., Ravendran P., Zhang J., Sankuratri S., Kondru R., Vauquelin G. (2014). A Study of
the Molecular Mechanism of Binding Kinetics and Long Residence Times
of Human CCR 5 Receptor Small Molecule Allosteric Ligands. Br. J. Pharmacol..

[26] Zia S. R., Coricello A., Bottegoni G. (2024). Increased Throughput in Methods for
Simulating Protein Ligand Binding and Unbinding. Curr. Opin. Struct. Biol..

[27] Sohraby F., Nunes-Alves A. (2023). Advances in
Computational Methods for Ligand Binding
Kinetics. Trends Biochem. Sci..

[28] Bernetti M., Masetti M., Rocchia W., Cavalli A. (2019). Kinetics of Drug Binding
and Residence Time. Annu. Rev. Phys. Chem..

[29] Bortolato A., Deflorian F., Weiss D. R., Mason J. S. (2015). Decoding the Role
of Water Dynamics in Ligand–Protein Unbinding: CRF _1_ R as a Test Case. J. Chem. Inf. Model..

[30] Deganutti G., Zhukov A., Deflorian F., Federico S., Spalluto G., Cooke R. M., Moro S., Mason J. S., Bortolato A. (2017). Impact of
Protein–Ligand Solvation and Desolvation on Transition State
Thermodynamic Properties of Adenosine A2A Ligand Binding Kinetics. In Silico Pharmacol..

[31] Capelli R., Bochicchio A., Piccini G., Casasnovas R., Carloni P., Parrinello M. (2019). Chasing the
Full Free Energy Landscape
of Neuroreceptor/Ligand Unbinding by Metadynamics Simulations. J. Chem. Theory Comput..

[32] Mahinthichaichan P., Liu R., Vo Q. N., Ellis C. R., Stavitskaya L., Shen J. (2023). Structure–Kinetics Relationships
of Opioids from Metadynamics
and Machine Learning Analysis. J. Chem. Inf.
Model..

[33] Galvani F., Pala D., Cuzzolin A., Scalvini L., Lodola A., Mor M., Rizzi A. (2023). Unbinding Kinetics of Muscarinic M3 Receptor Antagonists
Explained by Metadynamics Simulations. J. Chem.
Inf. Model..

[34] Potterton A., Husseini F. S., Southey M. W. Y., Bodkin M. J., Heifetz A., Coveney P. V., Townsend-Nicholson A. (2019). Ensemble-Based Steered Molecular
Dynamics Predicts Relative Residence Time of A _2A_ Receptor
Binders. J. Chem. Theory Comput..

[35] Deganutti G., Moro S., Reynolds C. A. (2020). A Supervised
Molecular Dynamics Approach
to Unbiased Ligand–Protein Unbinding. J. Chem. Inf. Model..

[36] Kokh D. B., Wade R. C. G. (2021). Protein-Coupled Receptor–Ligand
Dissociation
Rates and Mechanisms from Τramd Simulations. J. Chem. Theory Comput..

[37] Weinan E., Ren W., Vanden-Eijnden E. (2005). Finite Temperature String Method
for the Study of Rare Events. J. Phys. Chem.
B.

[38] Badaoui M., Buigues P. J., Berta D., Mandana G. M., Gu H., Földes T., Dickson C. J., Hornak V., Kato M., Molteni C., Parsons S., Rosta E. (2022). Combined Free-Energy
Calculation and Machine Learning Methods for Understanding Ligand
Unbinding Kinetics. J. Chem. Theory Comput..

[39] Buigues P. J., Gehrke S., Badaoui M., Dudas B., Mandana G., Qi T., Bottegoni G., Rosta E. (2023). Investigating the Unbinding of Muscarinic
Antagonists from the Muscarinic 3 Receptor. J. Chem. Theory Comput..

[40] Branduardi D., Gervasio F. L., Parrinello M. (2007). From A to
B in Free Energy Space. J. Chem. Phys..

[41] Capelli R., Lyu W., Bolnykh V., Meloni S., Olsen J. M. H., Rothlisberger U., Parrinello M., Carloni P. (2020). Accuracy of Molecular Simulation-Based
Predictions of *k*
_off_ Values: A Metadynamics
Study. J. Phys. Chem. Lett..

[42] Bernetti M., Masetti M., Recanatini M., Amaro R. E., Cavalli A. (2019). An Integrated
Markov State Model and Path Metadynamics Approach To Characterize
Drug Binding Processes. J. Chem. Theory Comput..

[43] Elisi G. M., Scalvini L., Lodola A., Mor M., Rivara S. (2022). Free-Energy
Simulations Support a Lipophilic Binding Route for Melatonin Receptors. J. Chem. Inf. Model..

[44] Casasnovas R., Limongelli V., Tiwary P., Carloni P., Parrinello M. (2017). Unbinding
Kinetics of a P38 MAP Kinase Type II Inhibitor from Metadynamics Simulations. J. Am. Chem. Soc..

[45] Grubmüller H. (1995). Predicting
Slow Structural Transitions in Macromolecular Systems: Conformational
Flooding. Phys. Rev. E:Stat. Phys., Plasmas,
Fluids, Relat. Interdiscip. Top..

[46] Voter A. F. (1997). Hyperdynamics:
Accelerated Molecular Dynamics of Infrequent Events. Phys. Rev. Lett..

[47] Tiwary P., Parrinello M. (2013). From Metadynamics
to Dynamics. Phys. Rev. Lett..

[48] Ray D., Ansari N., Rizzi V., Invernizzi M., Parrinello M. (2022). Rare Event Kinetics from Adaptive
Bias Enhanced Sampling. J. Chem. Theory Comput..

[49] Ansari N., Rizzi V., Parrinello M. (2022). Water Regulates
the Residence Time
of Benzamidine in Trypsin. Nat. Commun..

[50] Gobbo D., Piretti V., Di Martino R. M. C., Tripathi S. K., Giabbai B., Storici P., Demitri N., Girotto S., Decherchi S., Cavalli A. (2019). Investigating Drug–Target
Residence Time in
Kinases through Enhanced Sampling Simulations. J. Chem. Theory Comput..

[51] Mollica L., Decherchi S., Zia S. R., Gaspari R., Cavalli A., Rocchia W. (2015). Kinetics of Protein-Ligand Unbinding
via Smoothed Potential
Molecular Dynamics Simulations. Sci. Rep..

[52] Mollica L., Theret I., Antoine M., Perron-Sierra F., Charton Y., Fourquez J.-M., Wierzbicki M., Boutin J. A., Ferry G., Decherchi S. (2016). Molecular Dynamics Simulations and Kinetic Measurements to Estimate
and Predict Protein–Ligand Residence Times. J. Med. Chem..

[53] Callegari D., Lodola A., Pala D., Rivara S., Mor M., Rizzi A., Capelli A. M. (2017). Metadynamics Simulations Distinguish
Short- and Long-Residence-Time Inhibitors of Cyclin-Dependent Kinase
8. J. Chem. Inf. Model..

[54] Schuetz D. A., Bernetti M., Bertazzo M., Musil D., Eggenweiler H.-M., Recanatini M., Masetti M., Ecker G. F., Cavalli A. (2019). Predicting
Residence Time and Drug Unbinding Pathway through Scaled Molecular
Dynamics. J. Chem. Inf. Model..

[55] Kokh D. B., Amaral M., Bomke J., Grädler U., Musil D., Buchstaller H. P., Dreyer M. K., Frech M., Lowinski M., Vallee F., Bianciotto M., Rak A., Wade R. C. (2018). Estimation of Drug-Target
Residence Times by τ-Random
Acceleration Molecular Dynamics Simulations. J. Chem. Theory Comput..

[56] Ziada S., Diharce J., Raimbaud E., Aci-Sèche S., Ducrot P., Bonnet P. (2022). Estimation of Drug-Target Residence
Time by Targeted Molecular Dynamics Simulations. J. Chem. Inf. Model..

[57] Marchi M., Ballone P. (1999). Adiabatic Bias Molecular Dynamics:
A Method to Navigate
the Conformational Space of Complex Molecular Systems. J. Chem. Phys..

[58] Nunes-Alves A., Kokh D. B., Wade R. C. (2021). Ligand Unbinding Mechanisms and Kinetics
for T4 Lysozyme Mutants from τRAMD Simulations. Curr. Res. Struct. Biol..

[59] D’Arrigo G., Kokh D. B., Nunes-Alves A., Wade R. C. (2024). Computational Screening
of the Effects of Mutations on Protein-Protein off-Rates and Dissociation
Mechanisms by τRAMD. Commun. Biol..

[60] Ballesteros, J. A. ; Weinstein, H. [19] Integrated methods for the construction of three-dimensional models and computational probing of structure-function relations in G protein-coupled receptors. In Methods in Neurosciences; Elsevier, 1995; Vol. 25, pp 366–428. 10.1016/S1043-9471(05)80049-7.

[61] Haga K., Kruse A. C., Asada H., Yurugi-Kobayashi T., Shiroishi M., Zhang C., Weis W. I., Okada T., Kobilka B. K., Haga T., Kobayashi T. (2012). Structure
of the Human M2Muscarinic Acetylcholine Receptor Bound to an Antagonist. Nature.

[62] Kokh D. B., Doser B., Richter S., Ormersbach F., Cheng X., Wade R. C. (2020). A Workflow for Exploring Ligand Dissociation
from a Macromolecule: Efficient Random Acceleration Molecular Dynamics
Simulation and Interaction Fingerprint Analysis of Ligand Trajectories. J. Chem. Phys..

[63] Liu H., Hofmann J., Fish I., Schaake B., Eitel K., Bartuschat A., Kaindl J., Rampp H., Banerjee A., Hübner H., Clark M. J., Vincent S. G., Fisher J. T., Heinrich M. R., Hirata K., Liu X., Sunahara R. K., Shoichet B. K., Kobilka B. K., Gmeiner P. (2018). Structure-Guided Development
of Selective M3Muscarinic Acetylcholine Receptor Antagonists. Proc. Natl. Acad. Sci. U.S.A..

[64] Glossop P. A., Watson C. A. L., Price D. A., Bunnage M. E., Middleton D. S., Wood A., James K., Roberts D., Strang R. S., Yeadon M., Perros-Huguet C., Clarke N. P., Trevethick M. A., Machin I., Stuart E. F., Evans S. M., Harrison A. C., Fairman D. A., Agoram B., Burrows J. L., Feeder N., Fulton C. K., Dillon B. R., Entwistle D. A., Spence F. J. (2011). Inhalation by Design: Novel Tertiary
Amine Muscarinic
M _3_ Receptor Antagonists with Slow Off-Rate Binding Kinetics
for Inhaled Once-Daily Treatment of Chronic Obstructive Pulmonary
Disease. J. Med. Chem..

[65] Smith Z., Branduardi D., Lupyan D., D’Arrigo G., Tiwary P., Wang L., Krilov G. (2025). Towards Automated Physics-Based
Absolute Drug Residence Time Predictions. ChemRxiv.

[66] Wang L., Berne B. J., Friesner R. A. (2011). Ligand Binding to Protein-Binding
Pockets with Wet and Dry Regions. Proc. Natl.
Acad. Sci. U.S.A..

[67] Zia S. R., Gaspari R., Decherchi S., Rocchia W. (2016). Probing Hydration Patterns
in Class-A GPCRs via Biased MD: The A _2A_ Receptor. J. Chem. Theory Comput..

[68] Das S., Raucci U., Neves R. P., Ramos M. J., Parrinello M. (2024). Correlating
Enzymatic Reactivity for Different Substrates Using Transferable Data-Driven
Collective Variables. Proc. Natl. Acad. Sci.
U.S.A..

[69] Kistemaker L. E. M., Elzinga C. R. S., Tautermann C. S., Pieper M. P., Seeliger D., Alikhil S., Schmidt M., Meurs H., Gosens R. (2019). Second M _3_ Muscarinic
Receptor Binding Site Contributes to Bronchoprotection by Tiotropium. Br. J. Pharmacol..

[70] Kappel K., Miao Y., McCammon J. A. (2015). Accelerated Molecular Dynamics Simulations
of Ligand Binding to a Muscarinic G-Protein-Coupled Receptor. Q. Rev. Biophys..

[71] De
Vivo M., Masetti M., Bottegoni G., Cavalli A. (2016). Role of Molecular Dynamics
and Related Methods in Drug Discovery. J. Med.
Chem..

[72] Stegani B., Capelli R. (2025). Kinetic Rates Calculation via Non-Equilibrium Dynamics. arXiv.

[73] Schrödinger Release 2023-01; Maestro, Schrödinger, LLC, New York, NY, 2023.

[74] Gutiérrez-de-Terán H., Massink A., Rodríguez D., Liu W., Han G. W., Joseph J. S., Katritch I., Heitman L. H., Xia L., Ijzerman A. P., Cherezov V., Katritch V., Stevens R. C. (2013). The Role
of a Sodium Ion Binding Site in the Allosteric Modulation of the A2A
Adenosine G Protein-Coupled Receptor. Structure.

[75] Lu C., Wu C., Ghoreishi D., Chen W., Wang L., Damm W., Ross G. A., Dahlgren M. K., Russell E., Von Bargen C. D., Abel R., Friesner R. A., Harder E. D. (2021). OPLS4: Improving
Force Field Accuracy on Challenging Regimes of Chemical Space. J. Chem. Theory Comput..

[76] Friesner R. A., Banks J. L., Murphy R. B., Halgren T. A., Klicic J. J., Mainz D. T., Repasky M. P., Knoll E. H., Shelley M., Perry J. K., Shaw D. E., Francis P., Shenkin P. S. (2004). Glide:
A New Approach for Rapid, Accurate Docking and Scoring. 1. Method
and Assessment of Docking Accuracy. J. Med.
Chem..

[77] Schrödinger Release 2023-4: Glide; Schrödinger, LLC, New York, NY, 2023.

[78] Wu E. L., Cheng X., Jo S., Rui H., Song K. C., Dávila-Contreras E. M., Qi Y., Lee J., Monje-Galvan V., Venable R. M., Klauda J. B., Im W. (2014). CHARMM-GUI
Membrane Builder toward Realistic Biological Membrane Simulations. J. Comput. Chem..

[79] Decherchi S., Bottegoni G., Spitaleri A., Rocchia W., Cavalli A. (2018). BiKi Life
Sciences: A New Suite for Molecular Dynamics and Related Methods in
Drug Discovery. J. Chem. Inf. Model..

[80] Maier J. A., Martinez C., Kasavajhala K., Wickstrom L., Hauser K. E., Simmerling C. (2015). ff14SB: Improving
the Accuracy of
Protein Side Chain and Backbone Parameters from ff99SB. J. Chem. Theory Comput..

[81] Wang J., Wolf R. M., Caldwell J. W., Kollman P. A., Case D. A. (2004). Development
and Testing of a General Amber Force Field. J. Comput. Chem..

[82] Jorgensen W. L., Chandrasekhar J., Madura J. D., Impey R. W., Klein M. L. (1983). Comparison
of Simple Potential Functions for Simulating Liquid Water. J. Chem. Phys..

[83] Joung I. S., Cheatham T. E. (2008). Determination of Alkali and Halide Monovalent Ion Parameters
for Use in Explicitly Solvated Biomolecular Simulations. J. Phys. Chem. B.

[84] Bayly C. I., Cieplak P., Cornell W., Kollman P. A. (1993). A Well-Behaved Electrostatic
Potential Based Method Using Charge Restraints for Deriving Atomic
Charges: The RESP Model. J. Phys. Chem..

[85] Case, D. A. ; Aktulga, H. M. ; Belfon, K. ; Ben-Shalom, I. Y. ; Berryman, J. T. ; Brozell, S. R. ; Cerutti, D. S. ; Cheatham, T. E., III ; Cisneros, G. A. ; Cruzeiro, V. W. D. ; Darden, T. A. ; Duke, R. E. ; Giambasu, G. ; Gilson, M. K. ; Gohlke, H. ; Goetz, A. W. ; Harris, R. ; Izadi, S. ; Izmailov, S. A. ; Kasavajhala, K. ; Kaymak, M. C. ; King, E. ; Kovalenko, A. ; Kurtzman, T. ; Lee, T. S. ; LeGrand, S. ; Li, P. ; Lin, C. ; Liu, J. ; Luchko, T. ; Luo, R. ; Machado, M. ; Man, V. ; Manathunga, M. ; Merz, K. M. ; Miao, Y. ; Mikhailovskii, O. ; Monard, G. ; Nguyen, H. ; O’Hearn, K. A. ; Onufriev, A. ; Pan, F. ; Pantano, S. ; Qi, R. ; Rahnamoun, A. ; Roe, D. R. ; Roitberg, A. ; Sagui, C. ; Schott-Verdugo, S. ; Shajan, A. ; Shen, J. ; Simmerling, C. L. ; Skrynnikov, N. R. ; Smith, J. ; Swails, J. ; Walker, R. C. ; Wang, J. ; Wang, J. ; Wei, H. ; Wolf, R. M. ; Wu, X. ; Xiong, Y. ; Xue, Y. ; York, D. M. ; Zhao, S. ; Kollman, P. A. Amber 2022; University of California: San Francisco, 2022.

[86] Loncharich R. J., Brooks B. R., Pastor R. W. (1992). Langevin
Dynamics of Peptides: The
Frictional Dependence of Isomerization Rates of *N* -acetylalanyl- *N* ′-methylamide. Biopolymers.

[87] Berendsen H. J. C., Postma J. P. M., Van Gunsteren W. F., DiNola A., Haak J. R. (1984). Molecular
Dynamics with Coupling to an External Bath. J. Chem. Phys..

[88] Miyamoto S., Kollman P. A. (1992). Settle: An Analytical Version of the SHAKE and RATTLE
Algorithm for Rigid Water Models. J. Comput.
Chem..

[89] Darden T., York D., Pedersen L. (1993). Particle Mesh
Ewald: An *N* ·log­(*N*) Method
for Ewald Sums in Large Systems. J. Chem. Phys..

[90] The
PLUMED consortium (2019). Promoting
Transparency and Reproducibility in Enhanced Molecular Simulations. Nat. Methods.

[91] Izrailev S., Stepaniants S., Balsera M., Oono Y., Schulten K. (1997). Molecular
Dynamics Study of Unbinding of the Avidin-Biotin Complex. Biophys. J..

[92] Humphrey W., Dalke A., Schulten K. (1996). VMD: Visual Molecular
Dynamics. J. Mol. Graphics.

