# Supplementary material for: Adiabatic-Bias Molecular Dynamics Simulations Reveal the Impact of Mutations on Muscarinic Antagonist Unbinding Kinetics
Source: J Chem Inf Model. 2025 Jun 16;65(13):7129–42. doi: 10.1021/acs.jcim.5c00601 (PMC12264934; doi:10.1021/acs.jcim.5c00601)
Supplement: Supplementary file 1 [file ci5c00601_si_001.pdf]

Adiabatic-Bias Molecular Dynamics Simulations Reveal the Impact of Mutations on Muscarinic Antagonist Unbinding Kinetics

Adriana Coricello,[1] Anna Lisa Chiaravalle,[1] Maria Musgaard,[2] Benjamin Gerald Tehan,[2] Gian Marco Elisi,[1,] Giovanni Bottegoni[1,3,*]*

[1] Department of Biomolecular Sciences, Università degli Studi di Urbino Carlo Bo, Piazza Rinascimento 6, 61029, Urbino, Italy

[2] OMass Therapeutics Ltd, Building 4000, John Smith Dr, Oxford Business Park, ARC, Oxford OX4 2GX, United Kingdom

[3] Department of Pharmacy, University of Birmingham, Edgbaston, B15 2TT, Birmingham, United Kingdom

* Corresponding authors: Gian Marco Elisi (gianmarco.elisi@uniurb.it) and Giovanni Bottegoni (giovanni.bottegoni@uniurb.it).

Supplementary Figures and Tables

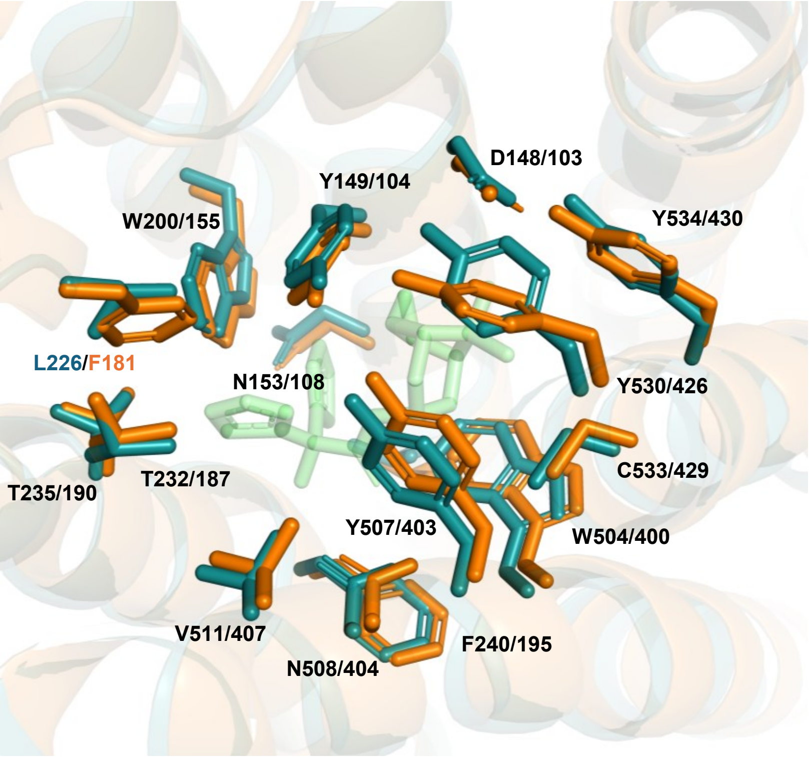

Figure S1. Representation of the orthosteric site residues of superimposed M3R (PDB ID 4DAJ) and M2R (PDB ID 3UON). Residues are represented as dark green and orange sticks, respectively. Protein backbones are represented as dark green (M3R) and orange (M2R) shaded cartoons. Tiotropium is bound to M3R and illustrated in lime green sticks. The only different residues directly facing the ligand are L226/F181 belonging to ECL2.

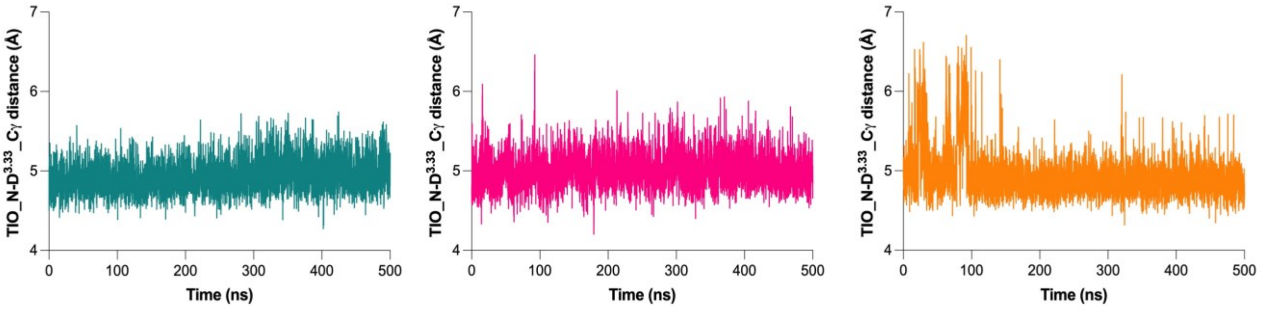

Figure S2. Plots of the distance between the tiotropium quaternary nitrogen atom and the D148/85$^{3.32}$ γ-carbon atom measured throughout unbiased MD simulations of WT and Y149A M3R (**A** and **B**), and M2R (**C**).

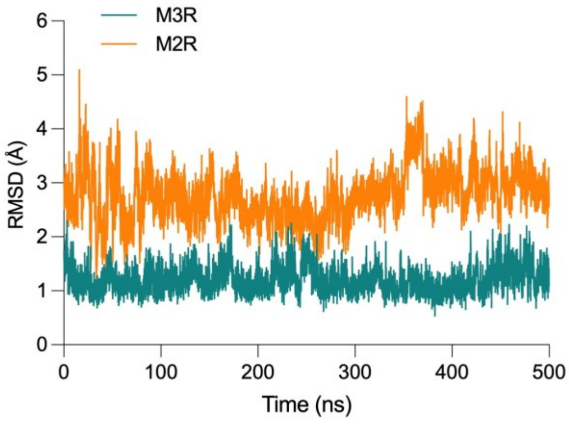

Figure S3. RMSD plot of the ECL2 residues C_α atoms throughout 500 ns of unbiased MD simulations of WT M3R (residues 212-223) and M2R (residues 167-184).

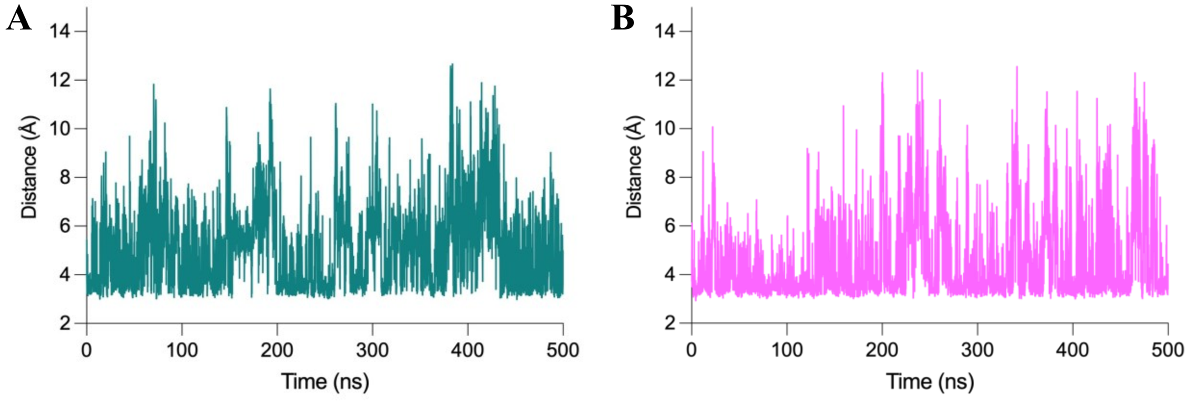

Figure S4. Plot of the distance between the K523$^{7.32}$ ε-nitrogen atom and the E220^{ECL2} γ-carbon atom calculated throughout 500 ns of unbiased MD simulations of WT M3R (**A**) and Y149A$^{3.33}$ (**B**).

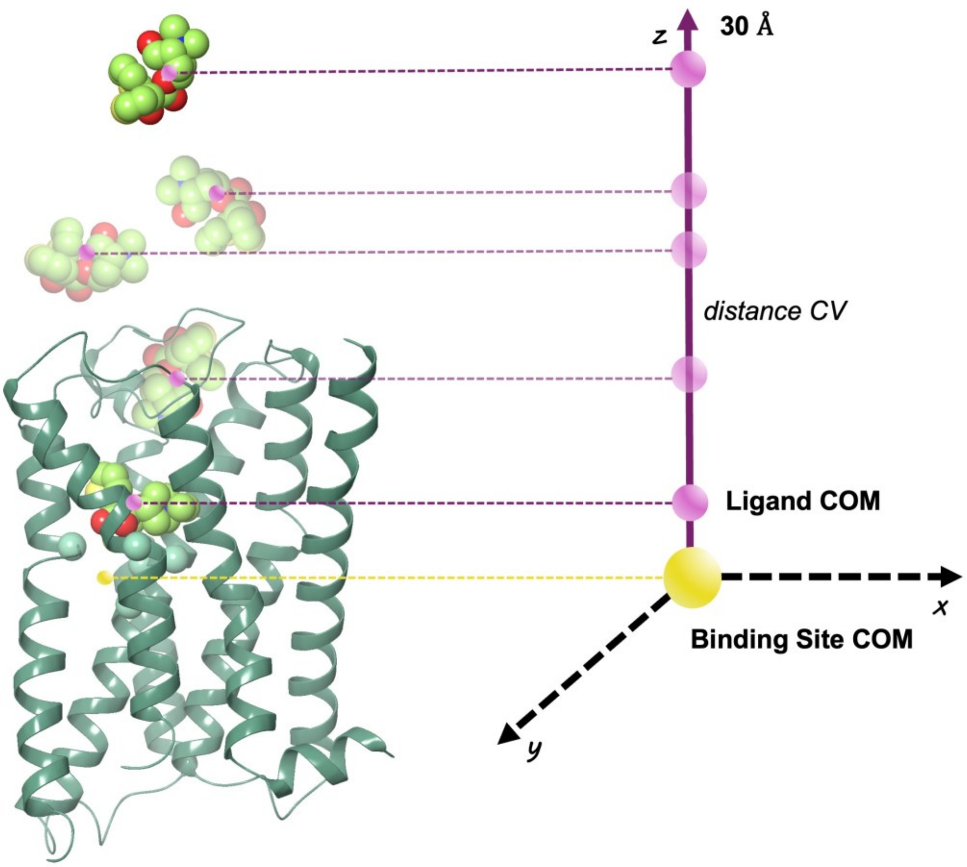

Figure S5. Schematic representation of the distance CV devised as the projection on the z-axis of the distance between the COM (pink and yellow spheres) of tiotropium (light green van der Waals spheres) and of the C_α atoms (dark green van der Waals spheres) of S152$^{3.36}$, N153$^{3.37}$, V156$^{3.40}$, A239$^{5.46}$, and W504$^{6.48}$.

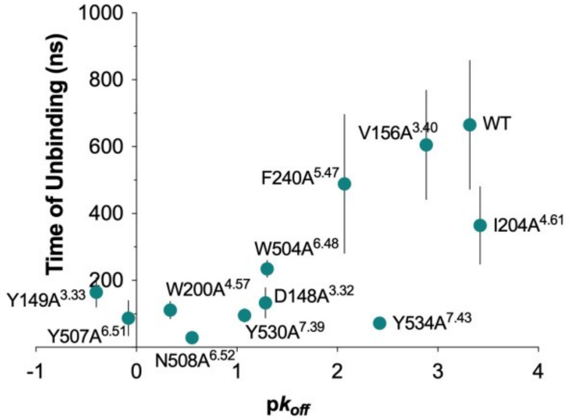

Figure S6. Plot of the experimental dissociation rates (pk_{off}) versus calculated mean residence times. The error bars represent the standard error of the mean of five replicas.

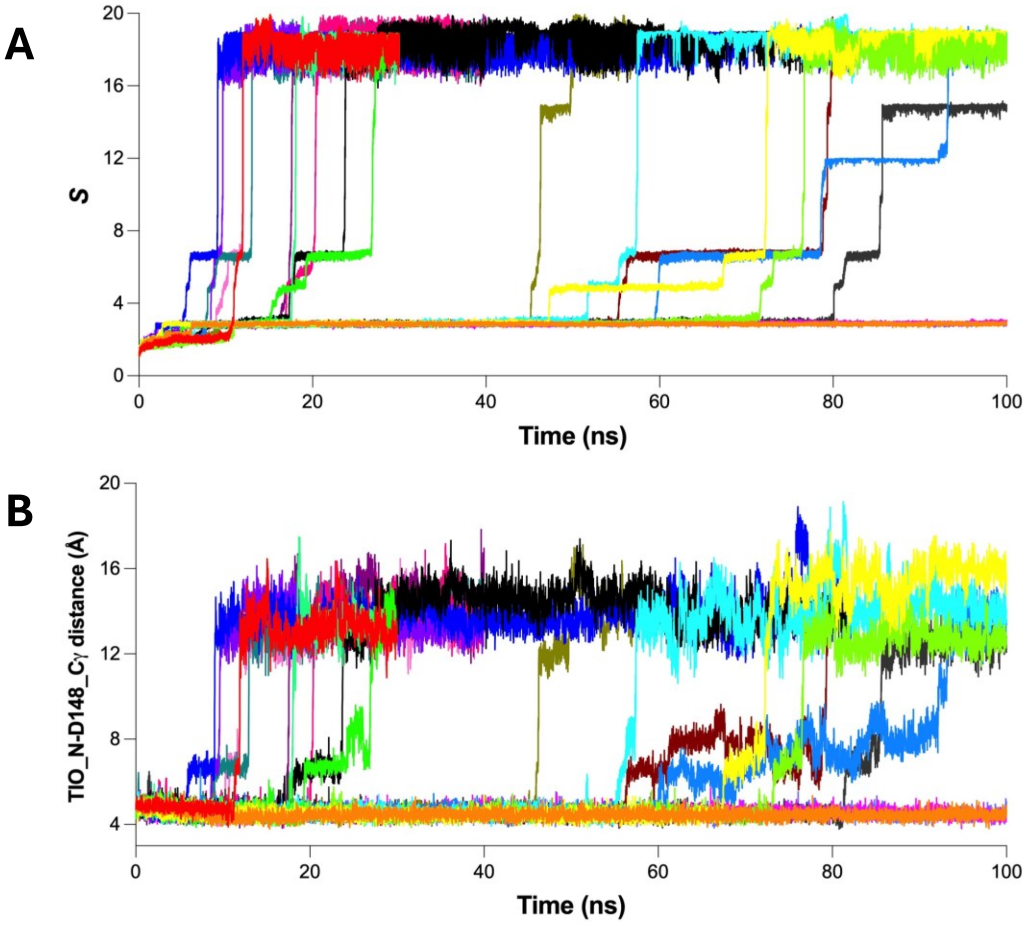

Figure S7. Plots of the path S (**A**) and of the distance between the tiotropium quaternary nitrogen atom and the D148$^{3.32}$ γ-carbon atom (**B**) throughout the 20 simulations of WT-M3R.

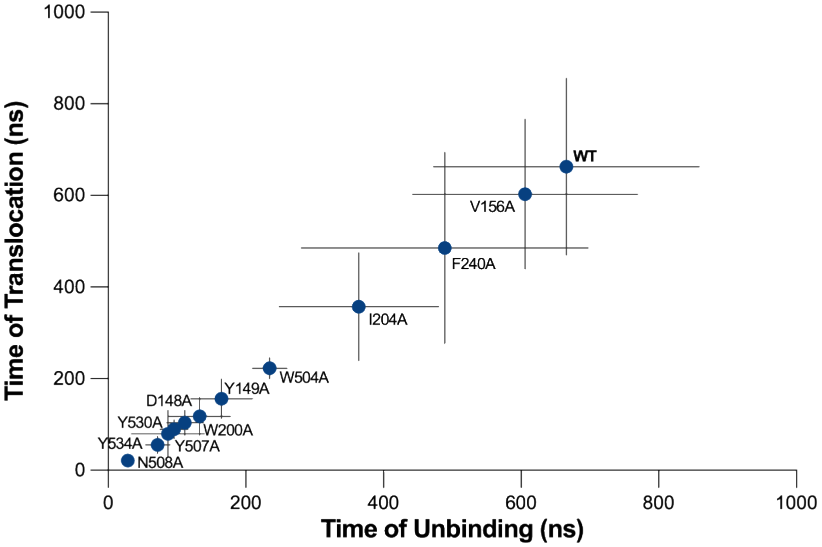

Figure S8. Plot of the translocation versus unbinding times calculated for the distance-ABMD dataset. The translocation was considered completed when tiotropium quaternary nitrogen had reached a distance of 12 Å from the Cγ atom of D148$^{3.32}$.

Table S1. Experimental dissociation rates (pk_{off}) versus mean translocation times calculated using PCV-ABMD. The times of translocation were defined as when the distance between the quaternary nitrogen atom and the D148$^{3.32}$ γ-carbon atom exceeds 12 Å.

	Experimental		Computed residence time	
**Mutant**	pk_{off}	Ranking	Translocation time ± SEM	Ranking
**I204A$^{4.61}$**	3.42	1	53.2 ± 9.7	3
**WT**	3.32	2	48.7 ± 8.0	4
**V156A$^{3.40}$**	2.89	3	84.2 ± 7.9	1
**Y534A$^{7.43}$**	2.42	4	31.1 ± 7.3	6
**F240A$^{5.47}$**	2.07	5	17.8 ± 5.2	8
**W504A$^{6.48}$**	1.30	6	54.9 ± 8.2	2
**D148A$^{3.32}$**	1.28	7	14.7 ± 5.6	10
**Y530A$^{7.39}$**	1.08	8	37.5 ± 8.2	5
**N508A$^{6.52}$**	0.55	9	17.2 ± 3.2	9
**W200A$^{4.57}$**	0.34	10	9.3 ± 2.8	12
**Y507A$^{6.51}$**	-0.08	11	20.8 ± 6.3	7
**Y149A$^{3.33}$**	-0.40	12	12.8 ± 4.1	11

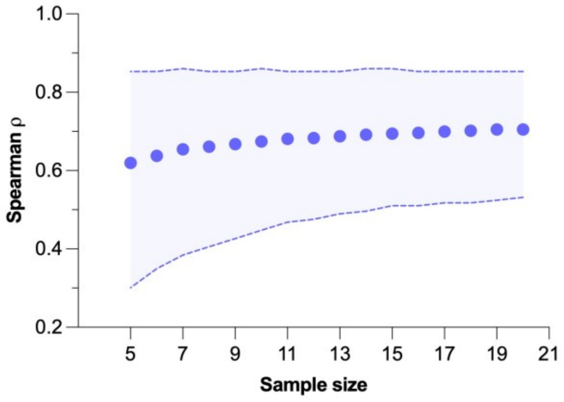

Figure S9. Plot of the mean Spearman correlations and the 95% confidence interval from the distribution of bootstrapped translocation times at different sample sizes. The times of translocation were defined as when the distance between the tiotropium quaternary nitrogen atom and the D148$^{3.32}$ γ-carbon atom exceeds 12 Å.

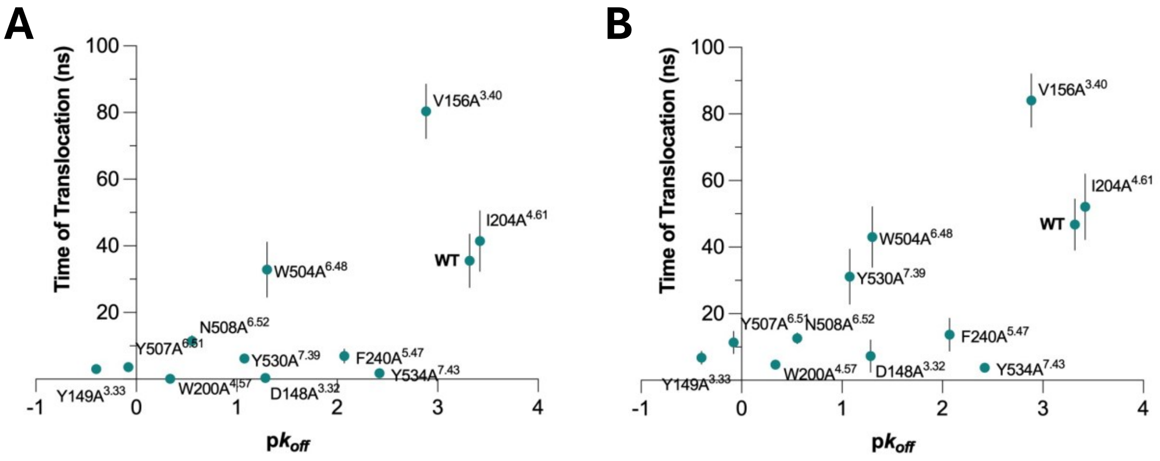

Figure S10. Plots of the experimental dissociation rates (pk_{off}) versus mean residence times calculated using the distance between the tiotropium quaternary nitrogen atom and the D148 γ-carbon atom at 6 (**A**) and 9 (**B**) Å as the figure of merit. The error bars represent the standard error of the mean of 20 replicas.

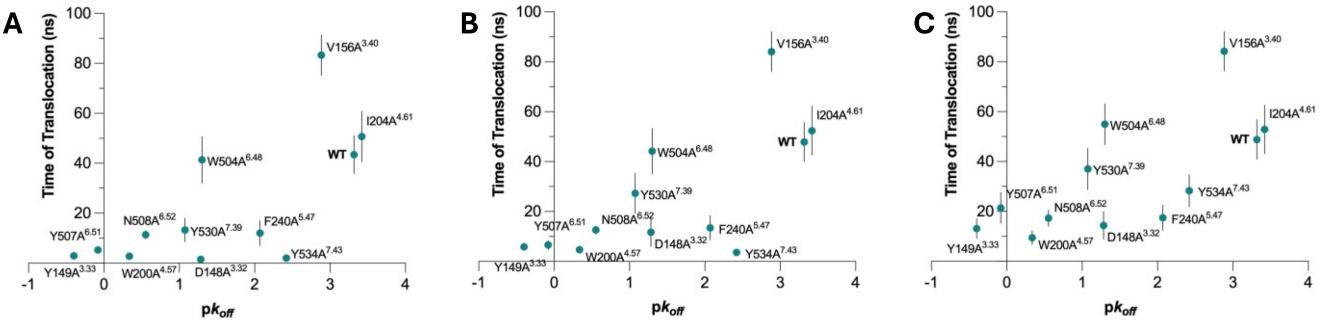

Figure S11. Plots of the experimental dissociation rates (pk_{off}) versus mean residence times calculated using the RMSD of the ligand with respect to the initial pose at 6 (**A**), 10 (**B**) and 14 (**C**) Å as the figure of merit. The error bars represent the standard error of the mean of 20 replicas.

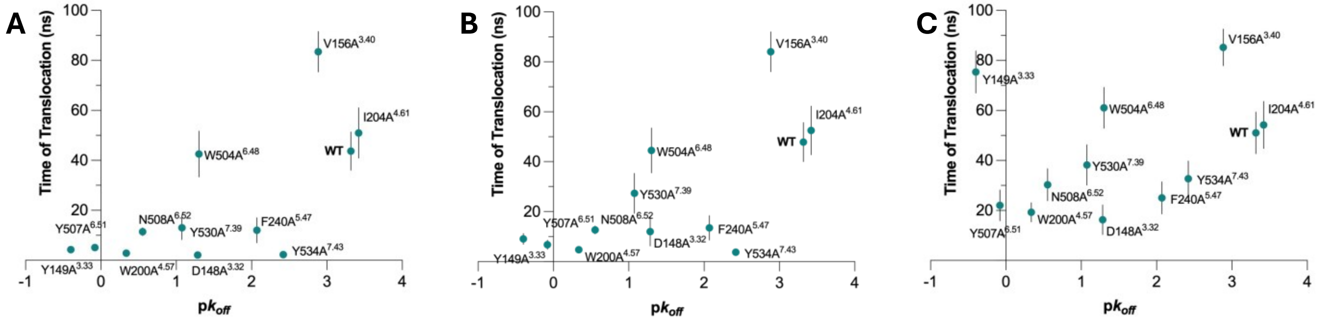

Figure S12. Plots of the experimental dissociation rates (pk_{off}) versus mean residence times calculated using the projection of the ligand-binding site COM distance along the *z* axis at 5 (**A**), 10 (**B**), and 15 (**C**) Å as the figure of merit. The error bars represent the standard error of the mean of 20 replicas.

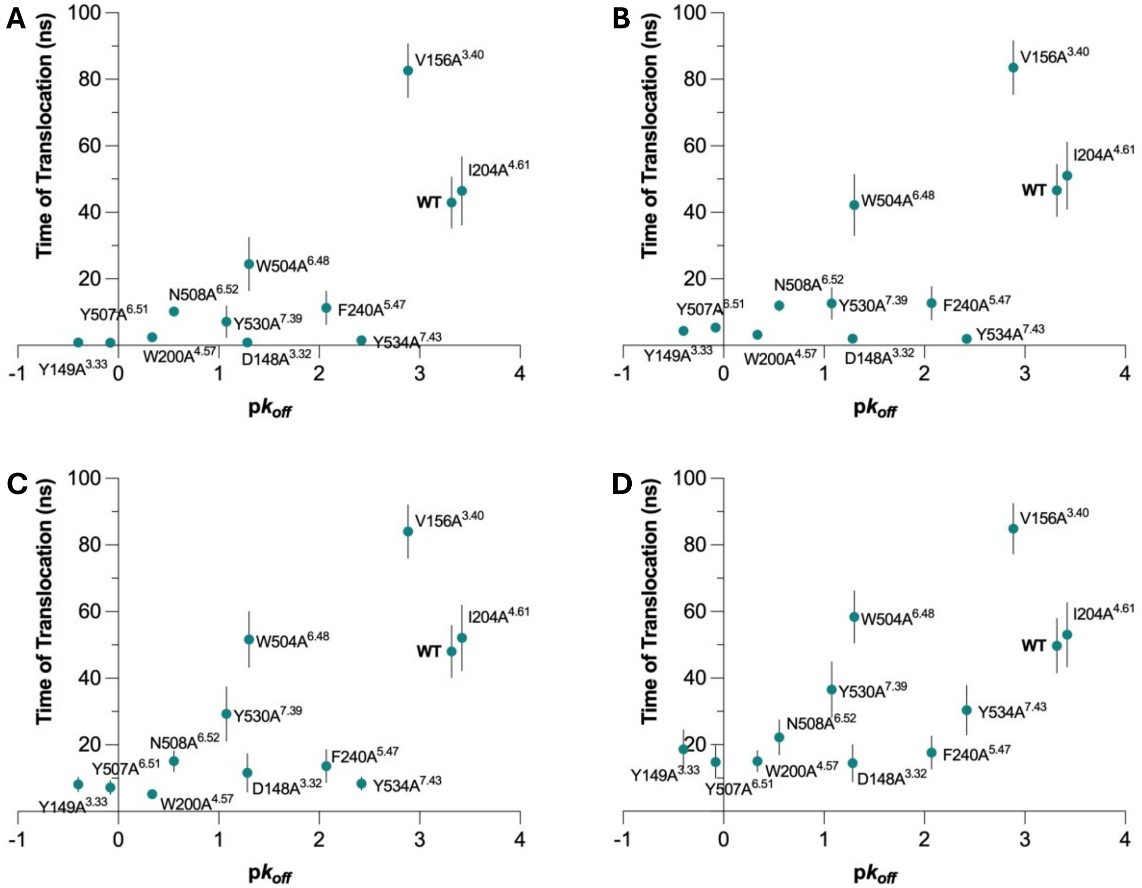

Figure S13. Plots of the experimental dissociation rates (pk_{off}) versus mean residence times calculated using the % SASA of the ligand at 10 % (**A**), 20 % (**B**), 30 % (**C**), and 40 % (**D**) as the figure of merit. The error bars represent the standard error of the mean of 20 replicas.

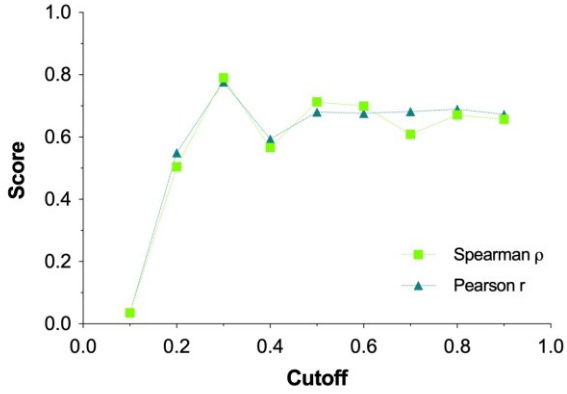

Figure S14. Plot of the Spearman and Pearson correlation coefficients obtained at different cutoffs of the Tanimoto distance of the IFP binary vectors calculated throughout the simulation.

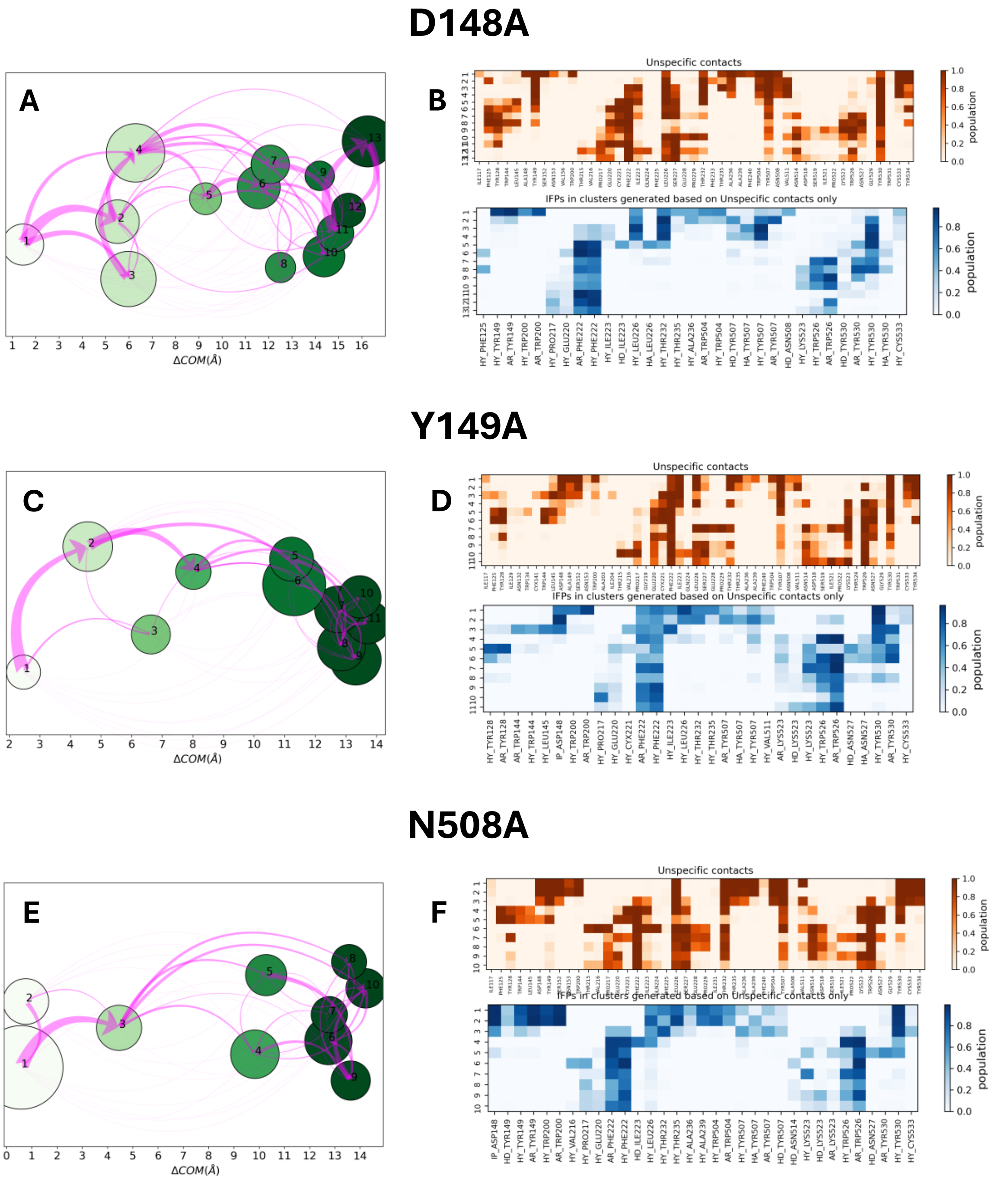

Figure S15. Schematic representation of the trajectory clustering analysis of the D148A$^{3.32}$ (**A** and **B**), Y149A$^{3.33}$ (**C** and **D**) and N508A$^{6.52}$ (**E** and **F**) systems. **A, C and E.** Clusters are shown by nodes positioned based on their ligand mean ΔCOM and the size is proportional to the cluster population. The green color of the nodes is darkened according to the increased averaged ligand RMSD. The magenta arrows indicate the total flow between two nodes. **B, D, and F.** IFP composition of each cluster. Nonspecific protein–ligand contacts and IFPs are represented in blue and orange maps, respectively.

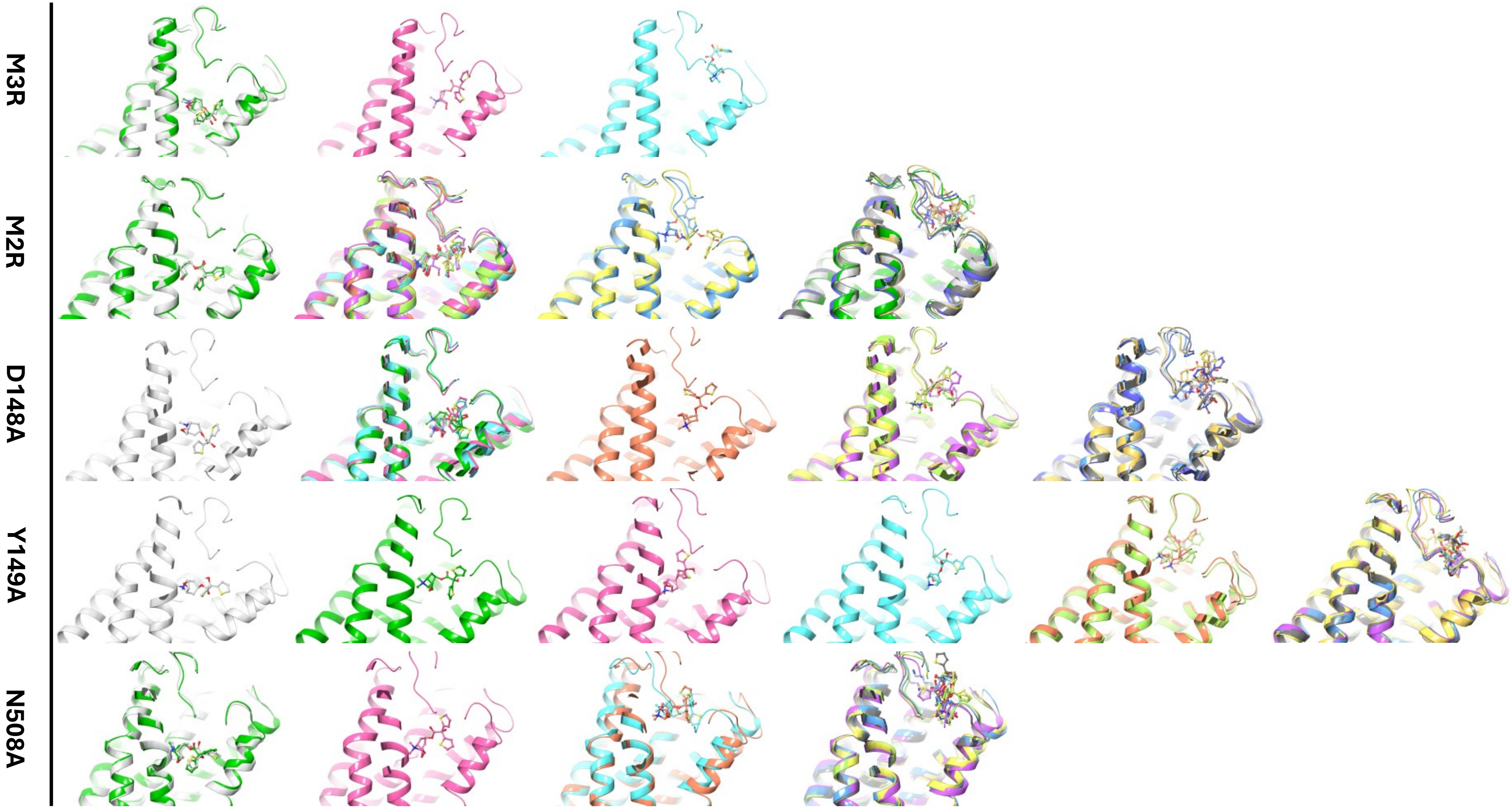

Figure S16. 3D representation of the cluster's centroids obtained through the analysis of the WT M3R, M2R, D148A$^{3.32}$, Y149A$^{3.33}$, and N508A$^{6.52}$ trajectories. Clusters are grouped according to their corresponding ligand RMSD with respect to the initial coordinates. The groups are represented as follows: WT: 1-2, 3, 4; hM2R: 1-2, 3-7, 8-9, 10-14; D148A: 1, 2-4, 5, 6-8, 9-14; Y149A: 1, 2, 3, 4, 5-6, 7-11; N508A: 1-2, 3, 4-5, 6-10.

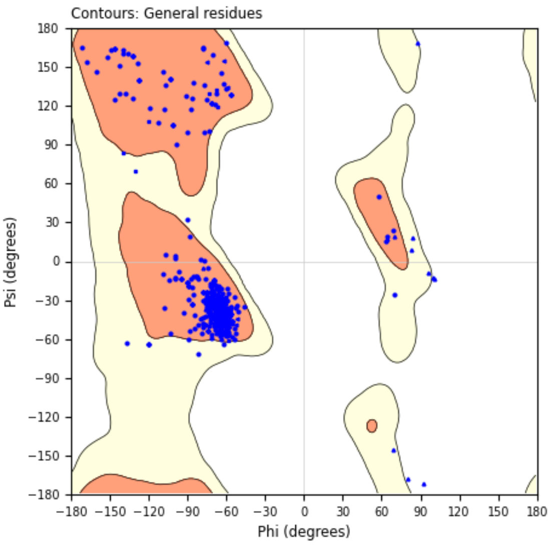

Figure S17. The Ramachandran plot of the minimized structure of the hM3R showed that all the backbone dihedral angles belong to permitted regions. The only residues with φ and ψ dihedral outside of this region are glycine residues.

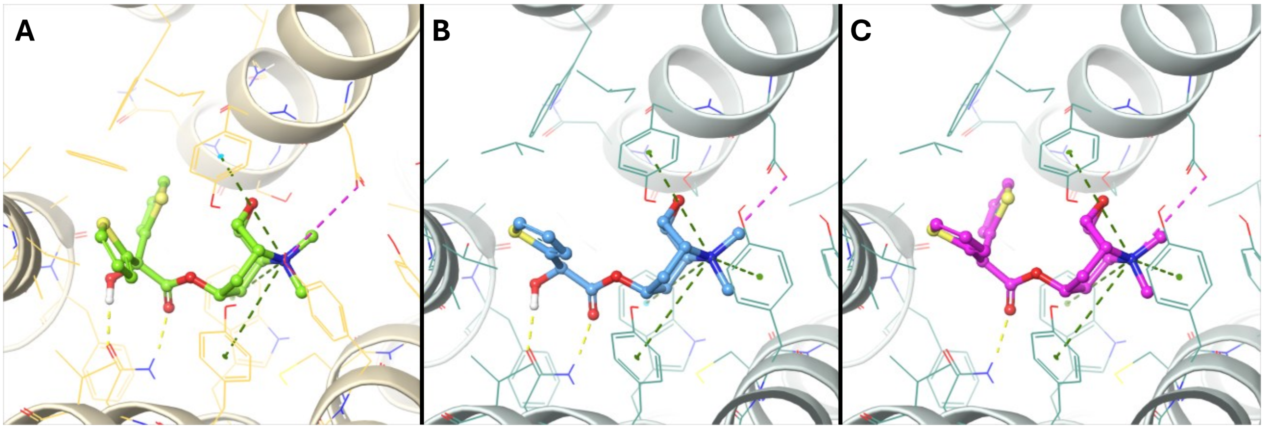

Figure S18. 3D representation of the docked poses of tiotropium (green sticks) within the orthosteric site of M2R (**A**, yellow ribbons), and of CPD1 (blue sticks) and CPD9 (pink sticks) in the binding site of M3R (**B** and **C**, teal ribbons). The ligands are shown in ball-and-stick, The proteins are shown as cartoon and the residues involved in crucial contacts with the compounds are reported as lines. Hydrogen bonds, salt bridges, and π-cation interactions are represented by yellow, magenta, and green dashed lines, respectively.

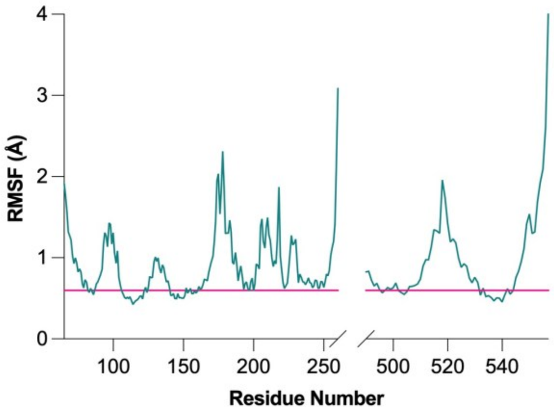

Figure S19. RMSF plot of WT-M3R C_α atoms calculated throughout 500 ns of unbiased MD simulation. The red line determines the 0.6 Å cut-off applied to select the α-carbons atoms for the alignment selection of the PCVs.

Methods Supplementary Information

Equilibration protocol

Prior to production, the systems were minimized for a maximum of 5,000 cycles, restraining the position of the protein and the ligand with a force constant of 10 kcal·mol^{-1}·Å^{-2} and of membrane lipids with a force constant of 2.5 kcal·mol^{-1}·Å^{-2}. Additional dihedral restraints were kept on lipid head groups. The system was then relaxed through different equilibration stages. The systems were heated from 100 to 303 K, during a timeframe of 250 ps, in the NVT ensemble and with a time-step of 0.001 ps. Subsequent system relaxation at the target temperature took place for 2.5 ns in NPT ensemble. Restraints were progressively decreased over both stages, scaling from 10.0 to 0.1 kcal·mol^{-1}·Å^{-2} for protein atoms, while dihedral restraints over lipid atoms were removed after 1 ns of the NPT phase. The thermalized systems were subsequently subjected to an additional step of 10 ns of unrestrained MD, with the same settings of the production runs.

Procedure for path generation and optimization

An initial guess path was built by binning and clustering the frames extracted from the preliminary unbinding simulations performed on the WT system in five distance ABMD replicas for an aggregated simulation time of nearly 4 μs. This procedure was managed using in-house VMD-Python scripts. The cumulative trajectory was firstly binned in seven 2 Å-wide histograms based on the *z*-axis component of the distance between the center of mass (COM) of tiotropium and the residues lining the floor of the binding site (Figure S5), until the ligand was displaced 12 Å from the original position. On each bin, a hierarchical clustering procedure was applied forming flat clusters based on a cophenetic distance not greater than 2. The centroid of each cluster was selected as cluster representative. The frames for the definition of the guess path were selected by picking the representatives of the most populated clusters of the seven bins.

Subsequently, the initial guess path consisting of seven frames was reparametrized using publicly available scripts employing a gradient-descent optimization of the MSD matrix. (1) The frameset was hence enriched through a steered molecular dynamics (SMD) simulation (2) of 50 ns using a force constant of 250 kcal·mol^{-1} along the $\mathcal{S}$ CV and in which the $\mathcal{Z}$ variable was walled at 6 Å^{-2}, and further optimized with the same reparameterization procedure, (1) resulting in a selection of twenty snapshots. An additional SMD of 50 ns and its optimization were performed to further refine the selection of the twenty frames. The last simulation was run using a force constant of 250 kcal·mol^{-1} along the $\mathcal{S}$ CV and in which the $\mathcal{Z}$ variable was walled at 10 Å^{-2}. The latter value was calibrated based on the exploration of the five distance ABMD replicas. The convergence of the final MSD matrix was assessed considering the metrics of the mean inter-frame distance (Figure S20). The analysis of frameset selection was performed according to a dedicated Plumed tutorial. (3)

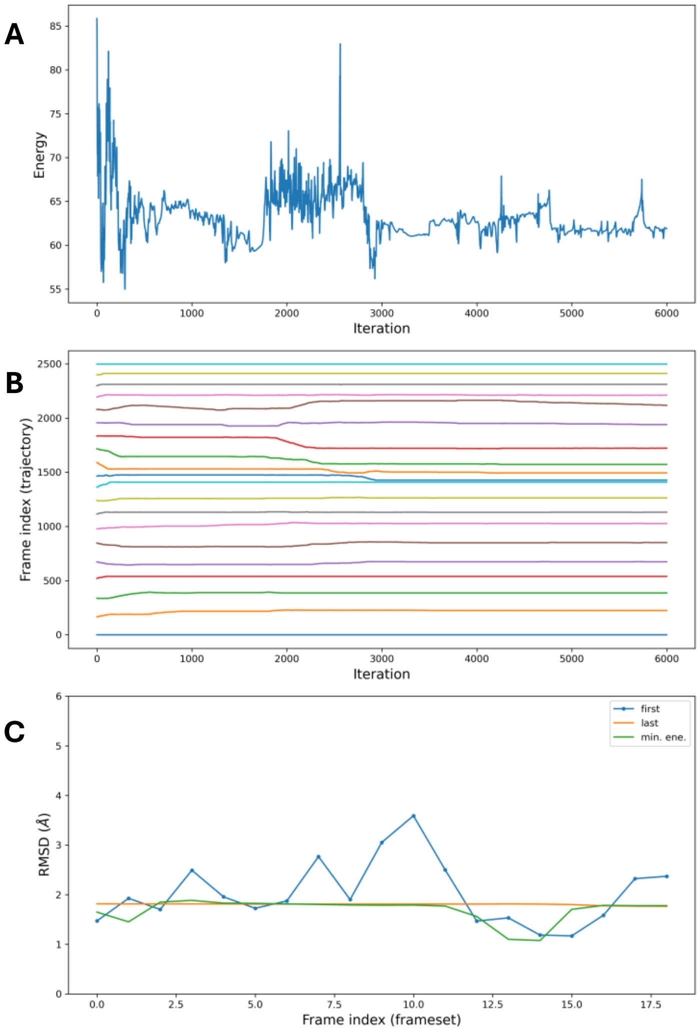

Figure S20. Convergence of the PCV optimization procedure over the number of iterations: plot of the energy associated with the gradient descent optimization (**A**); variation of frame selection during optimization (**B**); variation of RMSD between neighboring frames (**C**).

The same protocol reported for the WT system was attempted to generate PCVs for V156A$^{3.40}$ mutant and M2R, using λ values of 0.66 and 1.35 Å^{-2}, respectively. In the case of M2R, the quality of the frameset defining the PCVs and obtained through the first SMD was considered optimal, and another round of refinement was deemed unnecessary. Moreover, the definition of PCVs for M2R was kept coherent with that of the other systems by selecting for the alignment the α carbons corresponding to equivalent Ballesteros-Weinstein positions to those defined for M3R receptor (Figure S19).

Analysis of IFP-based metrics

IFPs and the subsequent clustering were calculated using the publicly available python MD-IFP workflow developed by Wade and coworkers. (4-5) The protocol is based on RDKit and the MDAnalysis tool and uses predefined IFPs to perform the analysis of trajectories. The contributions to the IFP are classified as hydrophobic (HY), aromatic (AR), hydrogen bond donor (HD) or acceptor (HA), salt bridge (IP/IN), halogen bonds (HL), and water bridge (WB). Also, nonspecific protein–ligand contacts are detected. The definitions of these classes are thoroughly described in Kokh et al. (6) Throughout the analysis, IFPs and unspecific contacts are extracted for each snapshot. For each independent replica, we calculated the Tanimoto distance between IFPs binary vectors of each frame with respect to the first one and used progressive cut-offs to generate estimations of the translocation times.

Clustering

Based on the unspecific contacts vectors, we performed clustering of each system using a step of 10 frames. For each replica, we selected the vectors corresponding to the frames with a ligand RMSD lower than 14 Å, plus 5 ns after this cut-off had been reached; moreover, replicas where the ligand RMSD never reached the cut-off were discarded. We hence performed k-means clustering on the merged replicas of each system. The optimal number of clusters for each system was estimated through a silhouette analysis (Figure S21). Finally, the clusters centroids were computed, and the hamming distance was employed to determine the closest points to the centroids and extract the coordinates of cluster representatives.

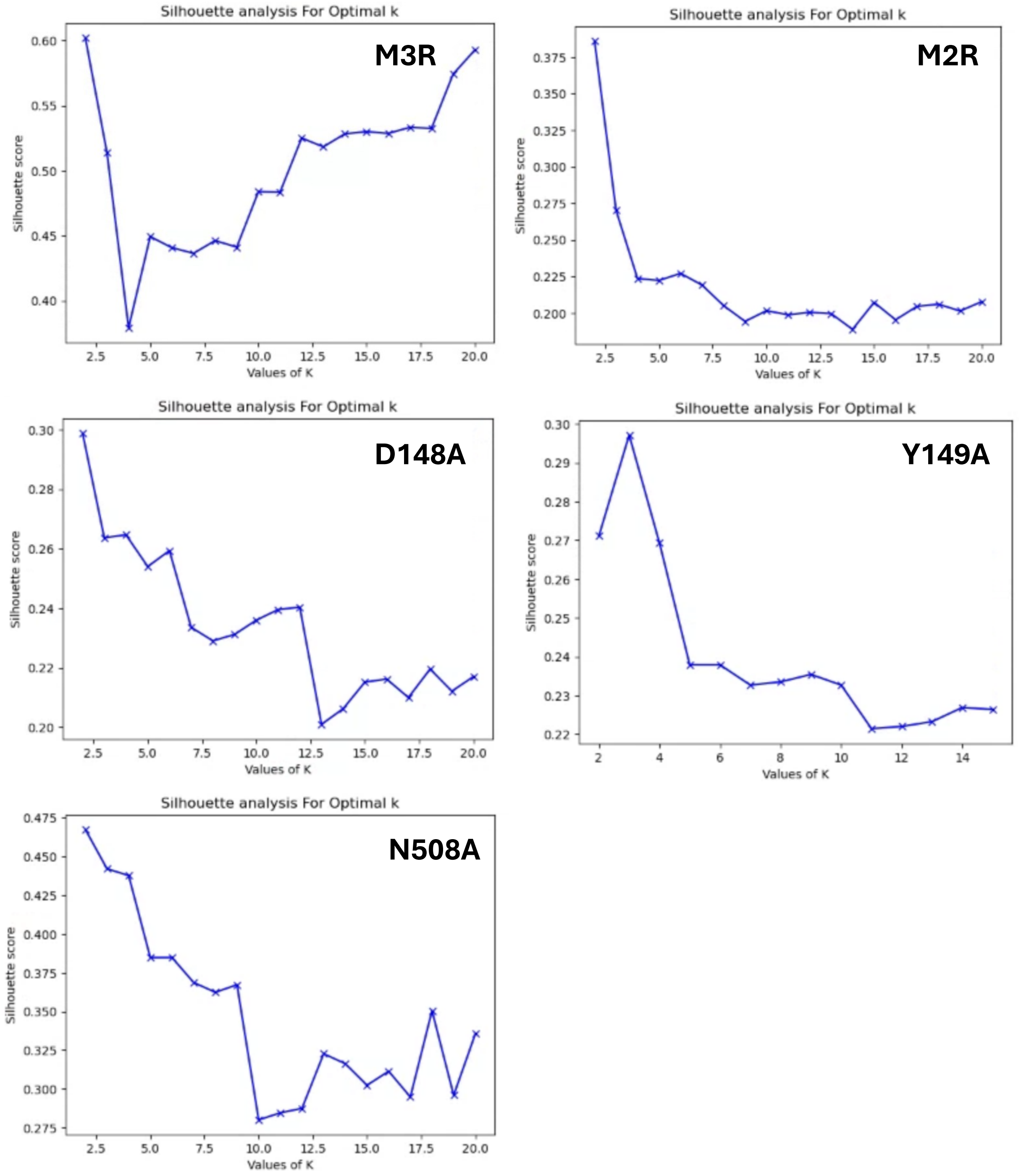

Figure S21. Plots of the silhouette scores obtained applying k-means clustering with different numbers of clusters.

References

(1) Scripts for selection of PCV frameset are available at https://github.com/davidebr/blog_material/tree/main/2_path_with_vmd.

(2) Izrailev, S.; Stepaniants, S.; Balsera, M.; Oono, Y.; Schulten, K. Molecular dynamics study of unbinding of the avidin-biotin complex. *Biophys J.* **1997**, 72, 1568–1581. https://doi.org/10.1016/S0006-3495(97)78804-0.

(3) Plumed tutorials can be found at http://www.plumed-tutorials.org/cite.html; https://arxiv.org/abs/2412.03595

(4) MD-IFP routine scripts are available at https://github.com/HITS-MCM/MD-IFp

(5) Kokh, D. B.; Wade, R. C. G Protein-Coupled Receptor–Ligand Dissociation Rates and Mechanisms from τRAMD Simulations. *J. Chem. Theory Comput.* **2021**, 17, 6610-6623, https://doi.org/10.1021/Acs.Jctc.1c00641.

(6) Kokh, D. B.; Doser, B.; Richter, S.; Ormersbach, F.; Cheng, X.; Wade, R. C. A Workflow for Exploring Ligand Dissociation from a Macromolecule: Efficient Random Acceleration Molecular Dynamics Simulation and Interaction Fingerprint Analysis of Ligand Trajectories. *J. Chem. Phys.* **2020**, *153* (12), 125102. https://doi.org/10.1063/5.0019088.
